# Inhibition of Erythromycin and Erythromycin-Induced Resistance among *Staphylococcus aureus* Clinical Isolates

**DOI:** 10.3390/antibiotics12030503

**Published:** 2023-03-02

**Authors:** Aya A. Mahfouz, Heba S. Said, Sherin M. Elfeky, Mona I. Shaaban

**Affiliations:** 1 Department of Microbiology and Immunology, Faculty of Pharmacy, Mansoura University, Mansoura 35516, Egypt; 2 Department of Microbiology and Immunology, Faculty of Pharmacy, Horus University-Egypt, New Damietta 34518, Egypt; 3 Department of Pharmaceutical Organic Chemistry, Faculty of Pharmacy, Mansoura University, Mansoura 35516, Egypt

**Keywords:** erythromycin resistance, inducible clindamycin resistance, MLS_B_, potential inhibitors

## Abstract

The increasing incidence of erythromycin and erythromycin-induced resistance to clindamycin among *Staphylococcus aureus* (*S. aureus*) is a serious problem. Patients infected with inducible resistance phenotypes may fail to respond to clindamycin. This study aimed to identify the prevalence of erythromycin and erythromycin-induced resistance and assess for potential inhibitors. A total of 99 isolates were purified from various clinical sources. Phenotypic detection of macrolide-lincosamide-streptogramin B (MLS_B_)-resistance phenotypes was performed by D-test. MLS_B_-resistance genes were identified using PCR. Different compounds were tested for their effects on erythromycin and inducible clindamycin resistance by broth microdilution and checkerboard microdilution methods. The obtained data were evaluated using docking analysis. Ninety-one isolates were *S. aureus*. The prevalence of constitutive MLS_B_, inducible MLS_B_, and macrolide-streptogramin (MS) phenotypes was 39.6%, 14.3%, and 2.2%, respectively. Genes including *ermC*, *ermA*, *ermB*, *msrA*, *msrB*, *lnuA*, and *mphC* were found in 82.6%, 5.8%, 7.7%, 3.8%, 3.8%, 13.5%, and 3.8% of isolates, respectively. Erythromycin resistance was significantly reduced by doxorubicin, neomycin, and omeprazole. Quinine, ketoprofen, and fosfomycin combated and reversed erythromycin/clindamycin-induced resistance. This study highlighted the significance of managing antibiotic resistance and overcoming clindamycin treatment failure. Doxorubicin, neomycin, omeprazole, quinine, ketoprofen, and fosfomycin could be potential inhibitors of erythromycin and inducible clindamycin resistance.

## 1. Introduction

*Staphylococcus aureus* (*S. aureus*) has been regarded as the most pathogenic bacterium of the Gram-positive *Staphylococcus* genus [1]. *S. aureus* is considered a commensal and opportunistic bacterium that colonizes the majority of the human population. It turns opportunistic when it invades populations with low immunity, such as immunocompromised people, diabetics, the elderly, and children, or when it enters the body through wounds [1,2]. *S. aureus* infections have been linked to high rates of morbidity and mortality. *S. aureus* is responsible for a wide range of diseases, from minor to moderate skin infections to more severe and even fatal infections, such as toxic shock syndrome, food poisoning, osteomyelitis, and endocarditis [3,4]. The multiple pathogenic implications of *S. aureus* are controlled by its potential virulence factors and the remarkable diversity of antibiotic resistance mechanisms [5]. Methicillin-resistant *S. aureus* (MRSA) isolates have been responsible for major outbreaks of nosocomial infections for many years and are now increasingly isolated from the community, where they can cause fatal infections [6].

Erythromycin, the first macrolide discovered in 1952, has been reported to be effective against a wide range of bacterial infections, including *S. aureus* [7,8]. Excessive use of erythromycin has led to the emergence of mechanisms responsible for its resistance. Several mechanisms have been involved in *S. aureus* resistance to erythromycin. The most common mechanism of resistance is the modification of the ribosomal binding site, which reduces erythromycin’s ability to bind to ribosomes. The synthesis of ribosomal methylase, which is encoded by *erm* genes, mediates this modification [9]. The active elimination of erythromycin by efflux systems, which are encoded by the *msr* and *mef* genes, is the second mechanism [10,11]. The third known cause of resistance in *S. aureus* is the production of macrolide-inactivating enzymes such as macrolide phosphorylases that are encoded by *mph* genes [12].

**Figure 1:**
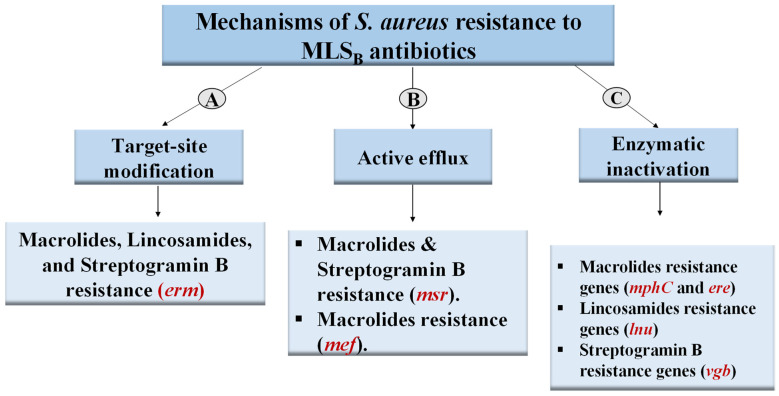
Mechanisms of *S. aureus* resistance to MLS_B_ antibiotics. MLS_B_: macrolides-lincosamides-streptogramin B.

Macrolides, such as erythromycin, lincosamides, such as clindamycin, and streptogramin B (quinupristin) are antibiotics that belong to a group collectively called the macrolides-lincosamides-streptogramin B (MLS_B_) group [13]. They are chemically and structurally different, but their mechanism of action is similar as they prevent bacterial protein synthesis by binding to 23s rRNA in the 50S ribosomal subunit [13,14]. MLS_B_ group is used to treat staphylococcal infections [13]. Three major mechanisms underlie *S. aureus* resistance to MLS_B_ (Figure 1): methylation of rRNA (target-site modification), active efflux, and enzymatic inactivation. In rRNA methylation, the methylase enzyme attaches one or two methyl groups to the adenine residue in the 23S rRNA moiety, lowering the affinity of the ribosomal subunit to MLS antibiotics [15]. The second reported mechanism is antibiotic efflux, which is mediated by *msr* genes, resulting in resistance to macrolide and streptogramin B antibiotics (MS) [16]. The third known mechanism is the enzymatic inactivation of antibiotics, which confers resistance to structurally related antibiotics only. For instance, specific resistance to macrolides is conferred by esterases and phosphotransferases, which are encoded by *ere* and *mphC* genes, respectively [17,18]. The *lnu* genes, which mediate resistance to only lincosamides, encode nucleotidyltransferases [19,20]. Furthermore, the *vgb* genes encode enzymes that hydrolyze streptogramin B, whereas the *vga* and *vat* genes confer resistance to streptogramin A [21].

MLS_B_-resistance phenotypes can be constitutive (cMLS_B_), in which rRNA methylase is usually produced. Inducible MLS_B_-resistance (iMLS_B_) phenotypes are elicited when rRNA methylase is produced in the presence of an inducing agent, such as erythromycin [14,22].

Infections caused by iMLS_B_ *S. aureus* isolates fail to respond to clindamycin therapy because the rRNA methylase enzyme secretion is active in the presence of erythromycin as an inducer, resulting in clindamycin inactivation and increasing antibiotic resistance. This prompts some researchers to warn against the use of clindamycin for *S. aureus* isolates with the iMLS_B_-resistance phenotypes [14,23,24].

The golden age of antibiotic therapy may be coming to an end due to the increasing incidence of antibiotic resistance worldwide. Resistance has an impact on all aspects of medicine and makes effective empirical therapy more challenging to achieve [25]. As a result, various inhibitor development strategies have been proposed and implemented. The enzymatic activity was exploited to search for compounds with potential inhibitory activity [26].

Previous studies on erythromycin combinations with pellitorine, sesamin, piperic acid and tetrahydropiperine [27], hydroxyamines [28], totarol [29], oleic and linoleic acids [30] showed significant decreases in minimum inhibitory concentration (MIC) of erythromycin against MS *S. aureus* isolates by inhibiting MsrA protein. Previous studies on CCCP [31], caffeine [32], piperine [33], omeprazole [34], vitamin D3 [35], vitamin K [36], verapamil [37], and digoxin [38] showed inhibitory activity on *S. aureus* efflux proteins. Neomycin also potentiated anti-staphylococcal activity of mupirocin [39]. Both meloxicam and ketoprofen were selected for their approved antimicrobial activity [40,41]. In addition, ketoprofen was reported to inhibit the methyltransferase enzyme responsible for the inactivation of thiopurine drugs [42]. Concerning quinine, a significant decrease was noted after combining quinine with other phytochemicals, such as reserpine [43]. Furthermore, a study reported in Australia demonstrated an additive effect between quinine and ampicillin [44]. Doxorubicin, 5-fluorouracil, and cisplatin have antimicrobial activity [45,46,47]. Additionally, doxorubicin was reported to inhibit DNA methyltransferase as a chemotherapeutic agent [48]. There are no previous studies approved the effect of potential inhibitors on the suppression of erythromycin resistance against iMLS_B_ *S. aureus* isolates. On the other hand, no previous studies were conducted to detect potential inhibitors of inducible clindamycin resistance.

The present study aims to determine the prevalence of resistance to different classes of antimicrobial agents, mainly the MLS_B_ group, among *S. aureus* isolated from different clinical sources. Moreover, the underlying mechanisms of MLS_B_-resistance phenotypes were explored. Furthermore, twenty-one compounds were selected to assess their inhibitory effects on erythromycin resistance and inducible clindamycin resistance. Potential inhibitors of resistance to erythromycin and inducible clindamycin were identified in order to overcome antibiotic resistance and achieve the desired therapeutic outcomes.

## 2. Results

### 2.1. Isolation and Identification of Isolates

Out of 99 staphylococcal isolates, 91 isolates were identified as *S. aureus* based on microscopical examination and biochemical reactions (D-mannitol fermentation and catalase and coagulase production). The isolates were collected from different hospitals near Mansoura University, Egypt. They were gathered from various clinical sources, including wounds (*n* = 36), blood (*n* = 30), nasal swabs (*n* = 24), and broncho-alveolar lavage (*n* = 1).

### 2.2. Antimicrobial Susceptibility Testing

**Figure 2:**
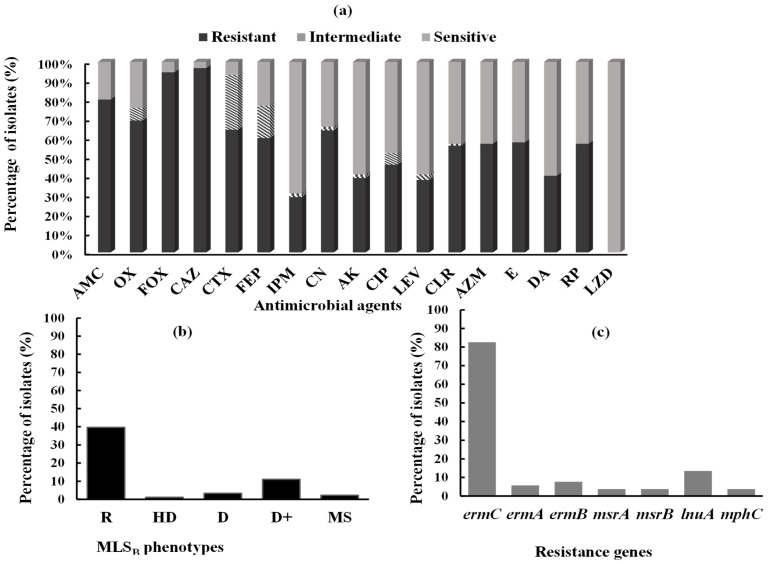
Antimicrobial resistance, phenotypic and genotypic detection of MLS_B_ determinants. (**a**) Prevalence of antimicrobial resistance among the tested *S. aureus* clinical isolates. AMC: amoxicillin-clavulanic acid, OX: oxacillin, FOX: cefoxitin, CAZ: ceftazidime, CTX: cefotaxime, FEP: cefepime, IPM: imipenem, CN: gentamicin, AK: amikacin, CIP: ciprofloxacin, LEV: levofloxacin, CLR: clarithromycin, AZM: azithromycin, E: erythromycin, DA: clindamycin, RP: quinapristin/dalfopristin, LZD: linezolid, MLS_B_: macrolide-lincosamide-streptogramin B. (**b**) Distribution of different MLS_B_ phenotypes among the tested *S. aureus* isolates. R: resistant to both erythromycin and clindamycin, HD: hazy D (HD) zone, two zones of growth around clindamycin disk, one zone with a light hazy growth up to clindamycin disk, and the second zone with heavy growth, showing D zone with two zones of growth around clindamycin disk, D: D zone positive with clear zone of D around clindamycin disk, D^+^: D zone positive with small colonies grew towards the clindamycin disk inside the D zone, MS: macrolide-streptogramin (MS) phenotype, resistant to erythromycin and sensitive to clindamycin without D zone. (**c**) Prevalence of MLS_B_-resistance genes among MLS_B_-resistant *S. aureus* isolates.

The susceptibility of the tested *S. aureus* clinical isolates (*n* = 91) to different classes of antimicrobial agents was detected. The isolates exhibited diverse resistance to tested antimicrobial agents, including ceftazidime (96.7%), cefoxitin (94.5%), amoxicillin-clavulanic acid (80.2%), oxacillin (69.2%), cefotaxime (64.8%), gentamicin (64.8%), cefepime (60.4%), ciprofloxacin (46.4%), levofloxacin (37.4%), clarithromycin (56%), azithromycin, erythromycin, quinapristin/dalfopristin (57.1%), clindamycin, amikacin (38.5%), and imipenem (28.6%). All isolates were sensitive to linezolid (Figure 2a, Table S1). Eighty-six isolates were identified as MRSA, where they showed resistance to cefoxitin and oxacillin (Table S1). The MIC of vancomycin among all isolates was less than 2 µg/mL. Therefore, no vancomycin-resistant *S. aureus* (VRSA) isolates were detected.

### 2.3. Phenotypic Detection of MLS_B_ Phenotypes

**Figure 3:**
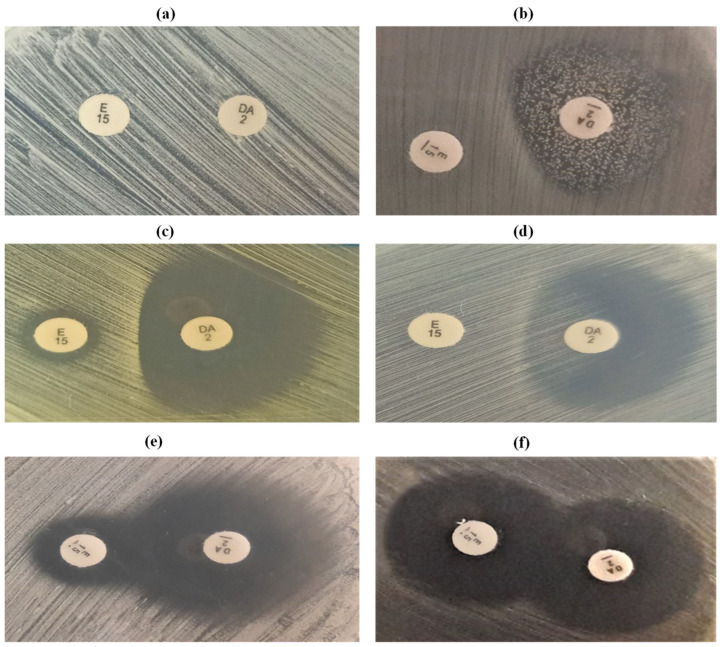
MLS_B_ phenotypes. (**a**) Isolate no. 47; R phenotype of constitutive MLS_B_ (cMLS_B_) in tested *S. aureus* isolates, resistant to both clindamycin and erythromycin. (**b**) Isolate no. 12; HD phenotype of cMLS_B_ in tested *S. aureus*, with two zones of growth around clindamycin disk, one zone with a light hazy growth up to clindamycin disk, and the second zone with heavy growth, showing D zone. (**c**) Isolate no. 10; D phenotype of inducible MLS_B_ (iMLS_B_), with a clear D zone around clindamycin disk. (**d**) Isolate no. 15; D^+^ phenotype of iMLS_B_, which revealed a D-shaped zone with small colonies growing towards the clindamycin disk inside the D zone. (**e**) Isolate no. 36; MS phenotype in tested *S. aureus* isolates that showed resistance to erythromycin and susceptibility to clindamycin without any D zone. (**f**) Isolate no. 27; S phenotype, sensitive to both clindamycin and erythromycin.

Different MLS_B_ phenotypes were observed among 91 *S. aureus* clinical isolates (Figure 2b). A total of 37 isolates exhibited cMLS_B_ resistance, in which 36 isolates showed the R phenotype; however, only one isolate showed the HD (hazy D zone) phenotype. The iMLS_B_ was detected in 13 isolates, where 3 isolates showed the D phenotype and 10 isolates showed the D^+^ phenotype. The MS phenotype was detected in two isolates. The remaining 39 isolates were sensitive to both erythromycin and clindamycin (S phenotype) (Figure 3).

### 2.4. Prevalence of MLS_B_-Resistance Genes

A total of 52 isolates with different MLS_B_-resistance phenotypes were screened for various resistant genes. PCR detection of *erm* genes (*ermA*, *ermB*, and *ermC*) revealed that the *ermC* gene was the most frequently detected gene; it was detected in 43 (82.6%) MLS_B_-resistant *S. aureus* isolates. The *ermA* and *ermB* genes were detected in three (5.8%) and four (7.7%) MLS_B_-resistant isolates, respectively. Additionally, PCR analysis revealed that *msrA* and *msrB* genes, which mediate the active efflux of macrolides and streptogramin B out of the cell, were found in two isolates, nos. 36 and 38 (3.8%) (Figure 2c, Table 1).

The *lnuA* gene, which confers specific resistance to lincosamides, was detected in seven isolates (13.5%). Additionally, isolates no. 36 and 38 (3.8%) were positive for the *mphC* gene that confers resistance to macrolides only (Figure 2c, Table 1).

### 2.5. Screening for the Effect of Different Compounds on Erythromycin Resistance

#### 2.5.1. Screening for the Effect of Different Compounds on Erythromycin Resistance against iMLS_B_ *S. aureus* Isolates

**Figure 4:**
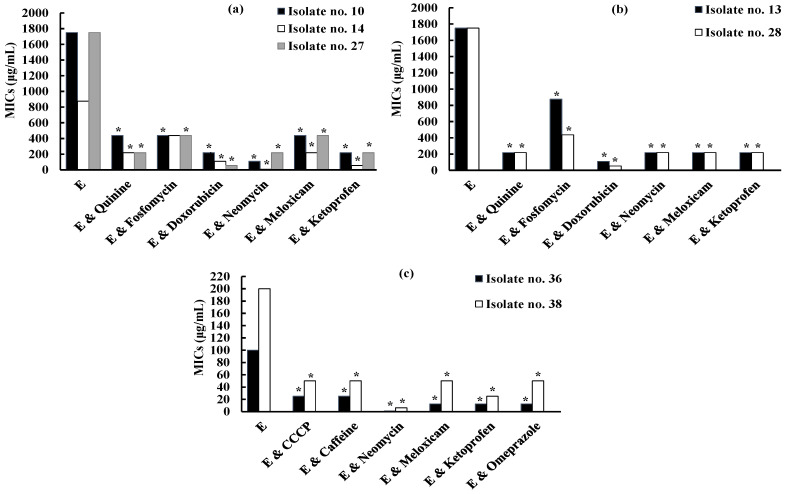
The effect of potential inhibitors on MIC of erythromycin among different MLS_B_ phenotypes of *S. aureus* isolates. (**a**) The effect of potential inhibitors (quinine, fosfomycin, doxorubicin, neomycin, meloxicam, and ketoprofen) on erythromycin resistance against D phenotype isolates no. 10, 14, and 27. (**b**) The effect of potential inhibitors (quinine, fosfomycin, doxorubicin, neomycin, meloxicam, and ketoprofen) on erythromycin resistance against D^+^ phenotype isolates no. 13 and 28. (**c**) The effect of potential inhibitors (CCCP, caffeine, neomycin, meloxicam, ketoprofen, and omeprazole) on erythromycin resistance against MS phenotype isolates no. 36 and 38. MICs: minimum inhibitory concentrations, E: erythromycin, CCCP: carbonyl cyanide m-chlorophenylhydrazine, *: probability-value (*p*-value) is <0.05, which is considered statistically significant.

The effect of tested compounds on erythromycin resistance against isolate no. 10 (D phenotype) showed that quinine, fosfomycin, and meloxicam decreased the MIC of erythromycin by 4-fold. Doxorubicin and ketoprofen reduced the MIC of erythromycin by 8-fold. Moreover, neomycin decreased the MIC of erythromycin by 16-fold. The remaining tested compounds showed only a 2-fold reduction in the MIC of erythromycin, while CCCP did not exhibit any effect on erythromycin (Table S2). Thus, quinine, fosfomycin, doxorubicin, neomycin, meloxicam, and ketoprofen were selected as potential inhibitors of erythromycin resistance against *S. aureus* isolate no. 10 and were similarly evaluated against other iMLS_B_ *S. aureus* isolates no. 14 and 27 (D phenotype) and no. 13 and 28 (D^+^ phenotype) (Figure 4, Table 2).

Quinine and meloxicam lowered the MIC of erythromycin by 4–8-fold against tested isolates. Upon the addition of fosfomycin, the MIC of erythromycin decreased by 2–4-fold against tested isolates. Doxorubicin decreased the MIC of erythromycin by 32-fold against tested isolates except isolates no. 14 and 13 (8–16-fold decrease). Moreover, neomycin achieved a reduction of 8-fold against all tested isolates except isolate no. 14 (128-fold decrease). Concerning ketoprofen, it showed an 8-fold decrease in erythromycin resistance against all isolates except isolate no. 14 (16-fold decrease) (Table 2).

#### 2.5.2. Screening for the Effect of Different Compounds on Erythromycin Resistance against MS *S. aureus* Isolates

The effect of different compounds on erythromycin resistance against isolate no. 36 indicated that neomycin exhibited a 128-fold decrease in the MIC of erythromycin. Meloxicam, ketoprofen, and omeprazole reduced the MIC of erythromycin by 8-fold, while CCCP and caffeine reduced the MIC of erythromycin by 4-fold. Compounds (quinidine, emetine, fosfomycin, ciprofloxacin, doxorubicin, and verapamil) showed only a 2-fold decrease in the MIC of erythromycin. On the other hand, other tested compounds showed no effect on erythromycin resistance (Table S3).

Therefore, CCCP, caffeine, neomycin, meloxicam, ketoprofen, and omeprazole were chosen as potential inhibitors of erythromycin resistance against *S. aureus* isolate no. 36 and were similarly assessed against other MS *S. aureus* isolate no. 38. Neomycin decreased the MIC of erythromycin by 32-fold. Ketoprofen showed an 8-fold reduction in the MIC of erythromycin. Furthermore, meloxicam, CCCP, caffeine, and omeprazole resulted in a 4-fold reduction in the MIC of erythromycin (Figure 4, Table 2).

### 2.6. Screening for the Effect of Different Compounds on Inducible Clindamycin Resistance against iMLS_B_ S. aureus Isolates

The MIC of clindamycin and tested compounds were determined against selected isolate no. 10 (D phenotype) (Table S4). Induction of clindamycin resistance using erythromycin (8 μg/mL) showed a 16-fold increase in the MIC of clindamycin (Table S4). The inhibitory activity of each tested compound on inducible clindamycin resistance was detected (Table S4). Quinine, fosfomycin, and ketoprofen showed a 4-fold decrease in the MIC of clindamycin and were selected to assess their effects against other iMLS_B_ *S. aureus* isolates no. 14 and 27 (D phenotype) and no. 13 and 28 (D^+^ phenotype) (Table S4).

Similarly, induction of clindamycin resistance by erythromycin (8 μg/mL) against *S. aureus* isolates no. 14 and 27 revealed a 16-fold increase in the MIC of clindamycin. Additionally, induction of clindamycin resistance using erythromycin against *S. aureus* isolates no. 13 and 28 showed a 256-fold increase in the MIC of clindamycin (Table 3).

Quinine showed a 4-fold decrease in the MIC of clindamycin against all tested isolates except isolate no. 13 (8-fold decrease). Additionally, the MIC of clindamycin was decreased by 4-fold after the addition of fosfomycin except isolate no. 27 (2-fold decrease). Moreover, a 4-fold reduction in the MIC of clindamycin was observed upon the addition of ketoprofen against all tested isolates, and more pronounced activity was observed with isolates no. 13 and 28 (32-fold decrease) (Table 3).

### 2.7. Checkerboard Microdilution Assay

Erythromycin/doxorubicin and erythromycin/neomycin combinations resulted in synergistic effects (fractional inhibitory concentration index (FICI) = 0.5) against isolate no. 10. Erythromycin with meloxicam, quinine, fosfomycin, and ketoprofen showed additive effects (Table 4). Synergistic effects were observed in erythromycin/neomycin and erythromycin/omeprazole combinations against isolate no. 36 with FICI = 0.313 and 0.5, respectively. Erythromycin combinations with CCCP, caffeine, meloxicam, and ketoprofen resulted in additive effects (Table 4).

Furthermore, the clindamycin/erythromycin combination produced an antagonistic effect with FICI = 16.668 against isolate no. 10. Quinine combinations with clindamycin and clindamycin/erythromycin showed additive effects. Moreover, fosfomycin revealed a synergistic effect when combined with clindamycin (FICI = 0.375). The clindamycin/erythromycin/fosfomycin combination exhibited an additive effect. Regarding ketoprofen, synergism was also detected in the clindamycin/ketoprofen combination (FICI = 0.5). The clindamycin/erythromycin/ketoprofen combination produced an additive effect (Table 5).

### 2.8. Molecular Docking Study for Potential Inhibitors of Erythromycin Resistance

#### 2.8.1. Molecular Docking Study of Doxorubicin at S-Adenosyl-L-Methionine (SAM)-Binding Site of ErmC’ Protein

**Figure 5:**
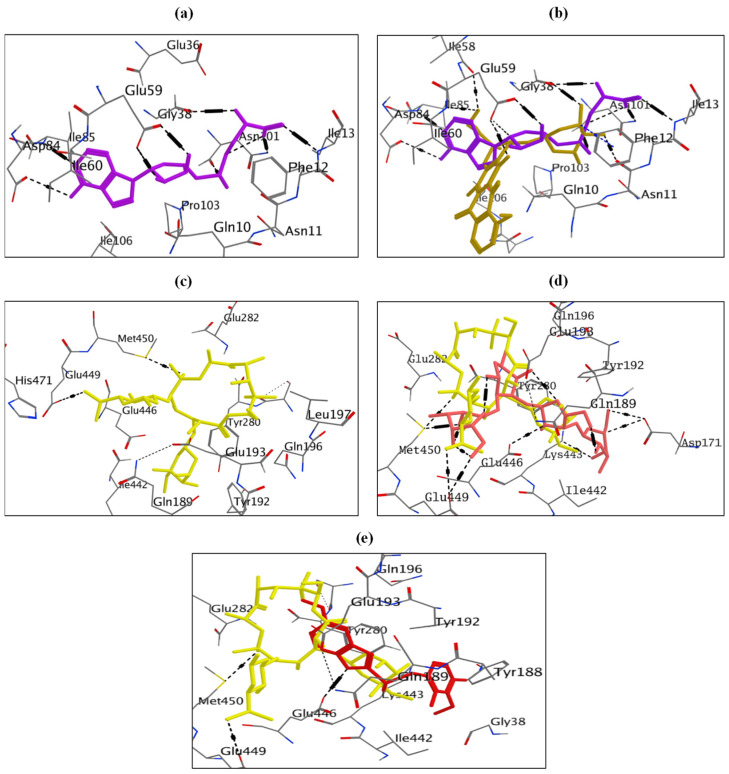
The molecular docking results of sinefungin (SFG) and doxorubicin at S-adenosyl-L-methionine (SAM) binding site of ErmC’ protein, and erythromycin, neomycin, and omeprazole at MsrA protein. (**a**) 3D interaction of SFG (violet) at SAM-binding site of ErmC’. (**b**) 3D diagram of overlay view of doxorubicin (dark yellow) and SFG (violet) at SAM-binding site of ErmC’. (**c**) 3D representation of erythromycin (yellow) at active site of MsrA protein. (**d**) 3D representation of overlay view of neomycin (dark pink), erythromycin (yellow) at active site of MsrA protein. (**e**) 3D representation of overlay view of omeprazole (red) and erythromycin (yellow) at active site of MsrA protein. For clarity, non-interacting residues were deleted. Hydrogen bonds are shown in black, and arene hydrogen bonds are shown in dark blue (UniProtKB ID: Q9ZNK9).

The docking of doxorubicin at ErmC’ showed a docking energy score = −7.59 kcal/mol, and it was capable of binding similarly to the binding mode of the co-crystallized ligand (sinefungin) (−9.75 kcal/mol), which included six hydrogen bonding interactions, one *H*-π interaction with Phe12, and ten hydrophobic interactions (Figure S1). Two hydrogen bonds with Glu38 and Glu59 and all hydrophobic interactions, except those with Ile85, Asp61, and Ser9, were common in doxorubicin and sinefungin (Figure 5, Table S5).

#### 2.8.2. Molecular Docking Study of Neomycin and Omeprazole at Binding Site of MsrA Protein

From the docking study, neomycin showed a binding affinity of −7.21 kcal/mol compared to erythromycin, which showed a docking score of −5.91 kcal/mol. Neomycin formed six hydrogen bonds, an *H*-Pi interaction with Tyr280, an ionic bonding interaction with Glu193, and eight hydrophobic interactions (Figure S1). Neomycin and erythromycin showed similar interactions at the same binding site of MsrA protein, forming *H*-bonding with Glu449 and Met450 and hydrophobic interactions with Glu446, Met450, Tyr280, Gln189, Tyr192, Ile442, and Glu193 residues (Figure 5, Table S6).

Omeprazole, on the other hand, was capable of docking at the MsrA protein as well, where it formed an *H*-bond interaction with Glu446 and nine hydrophobic interactions (Figure S1). It showed a docking score of −6.93 kcal/mol. Additionally, omeprazole showed similar bonds with erythromycin at the binding site of MsrA protein, where both compounds bind by hydrogen bonding interaction with Glu446, in addition to hydrophobic interactions with Glu446, Tyr280, Tyr192, Ile442, and Glu193 (Figure 5, Table S6).

### 2.9. Molecular Docking Study of Quinine, Fosfomycin and Ketoprofen at SAM-Binding Site of ErmC’ Protein

**Figure 6:**
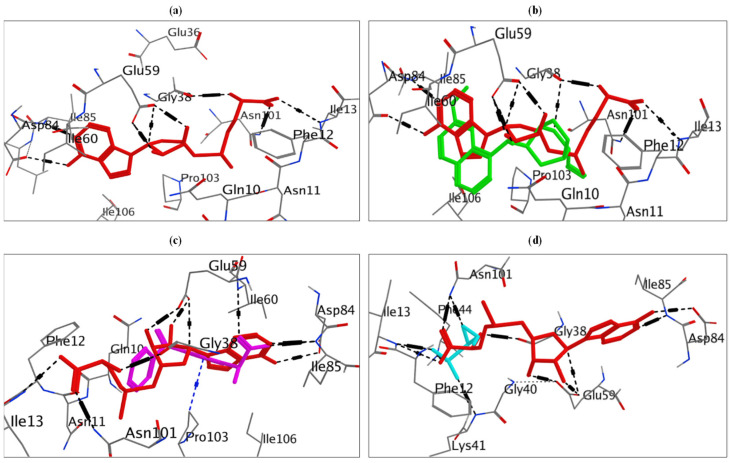
The molecular docking results of SAM, quinine, ketoprofen, and fosfomycin at the SAM-binding site of ErmC’ protein. (**a**) 3D diagram of SAM (red) at SAM-binding site of ErmC’. (**b**) 3D diagram of overlay view of quinine (green) and SAM (red) at SAM-binding site of ErmC’. (**c**) 3D diagram of overlay view of ketoprofen (magenta) and SAM (red) at SAM-binding site of ErmC’. (**d**) 3D diagram of overlay view of fosfomycin (cyan) and SAM (red) at SAM-binding site of ErmC’. For clarity, non-interacting residues were deleted. Hydrogen bonds are shown in black, and arene hydrogen bonds are shown in dark blue (PDB ID: 1QAO).

Quinine showed a consistent binding mode (docking energy score (ΔG) = −6.87 kcal/mol) to the co-crystallized ligand (SAM) (docking energy score (ΔG) = −8.58 kcal/mol), including two hydrogen bonding interactions with Glu59 and Gly38 residues of ErmC’, one ionic bonding interaction of amino group in quinine with Glu59, and seven hydrophobic interactions (Figure S2). Moreover, quinine and SAM can fit the SAM-binding site of ErmC’. It showed similar hydrogen bond interactions (with Glu59 and Gly38) and hydrophobic interactions when compared to SAM (Figure 6, Table S7).

On the other hand, molecular modeling studies showed that ketoprofen had a binding mode (docking energy score (ΔG) = −6.52 kcal/mol) compared to SAM (docking energy score (ΔG) = −8.58 kcal/mol). Ketoprofen interacted with Ile60 and Ile85 residues by hydrogen bonds; it also interacted by Pro103 by an *H*-Pi interaction and demonstrated five hydrophobic interactions (Figure S2). Ketoprofen and SAM fit the same binding site and showed similar interactions with Ile85 (*H*-bond) and all hydrophobic interactions except those with Asn11 and Asn101 (Figure 6, Table S7).

Docking studies also showed the involvement of five hydrogen bonds with fosfomycin, with a docking energy score (ΔG) of −4.19 kcal/mol when compared to SAM (−8.58 kcal/mol) (Figure S2). Ile13 and Asn101 appeared to form common hydrogen bonds in fosfomycin and SAM (Figure 6, Table S7).

## 3. Discussion

*Staphylococcus aureus* is an opportunistic Gram-positive pathogen that causes a variety of clinical infections [49]. It is commonly isolated from infections in surgical sites, chronic ulcers, purulent cellulitis, and wounds [50]. It can spread throughout the body and affect organs, such as the brain, kidneys, hearts, muscles, bones, eyes, joints, and lungs [51].

In this study, 91 *S. aureus* isolates were collected from different clinical sources. Wounds were the most common source of infection (39.6%), followed by blood (33%) and nasal swabs (26.4%). Broncho-alveolar lavage was the least common type of specimen (1.1%). This finding is similar to that reported by Razeghi et al. [52], where wounds were the major source (43.4%), followed by blood (18.1%), among 490 *S. aureus* clinical isolates.

The antimicrobial susceptibility test demonstrated a high level of resistance to β-lactam antibiotics, such as amoxicillin-clavulanic acid (80%), oxacillin (69.2%), cefoxitin (94.5%), ceftazidime (96.7%), cefotaxime (64.8%), and cefepime (60.4%). A moderate level of resistance was observed towards aminoglycoside antibiotics, such as gentamicin (64.8%) and amikacin (38.5%), fluoroquinolones, such as ciprofloxacin (46.2%) and levofloxacin (37.4%), macrolides, such as erythromycin and azithromycin (57.1%), and clarithromycin (56%). Concerning clindamycin and quinapristin/dalfopristin, the resistance rates were 38.5% and 57.1%, respectively. The most effective antibiotics were linezolid and vancomycin (100%) and imipenem (71.4%) for the management of infections caused by *S. aureus* (Figure 2a, Table S1).

In the current study, 94.5% of *S. aureus* isolates were MRSA (Table S1). Similarly, a high frequency of MRSA has also been reported in Egypt (89.4%) [53], Peru (80%) [54], and Colombia (90%) [55]. Among *S. aureus* isolates collected in this study, 63.7% were multidrug-resistant (MDR) (Table S1), which is similar to the rate reported in Egypt (63%) [56].

The prevalence of cMLS_B_, iMLS_B_ and MS resistance phenotypes among *S. aureus* isolates was 39.6%, 14.3%, and 2.2%, respectively (Figure 2b and Figure 3). A comparable rate of cMLS_B_ was found by Kishk and co-authors (38.6%) [57]. The iMLS_B_ prevalence is comparable to that reported in Egypt (13.64%) [57], Nepal (11.48%) [58], India (22.0%) [59], and Malaysia (22.1%) [60]. The incidence of the MS phenotype among our isolates is in accordance with that previously reported in Egypt (2.27%) [57], Jordan (2.82%) [61], Greece (2.90%) [62], and Ethiopia (1.26%) [63].

In this study, the inducible MLS_B_ mechanism was less frequent than the constitutive mechanism, but both were associated with the presence of the *erm* genes. Target-site modification is encoded by *erm* genes that cause the methylation of 23S rRNA, resulting in resistance to the MLS_B_ group [64]. The predominant gene among iMLS_B_ isolates was the *ermC* gene (*n =* 10/13; 76.9%), followed by *ermA* (*n* = 2/13; 15.4%) and *ermB* (*n* = 1/13, 7.7%) (Table 1). In accordance with our findings, other studies reported the prevalence of the *ermC* gene among iMLS_B_ isolates [61,65,66,67].

Compared to *erm* genes, lower percentages of *msr*-carrying isolates were identified (3.8%). The *msr* genes mediate the active efflux of macrolides and streptogramin B and reduce their intracellular concentrations [14]. Isolates containing the *msr* genes encoded the MS resistance phenotype and harbored the *mphC* gene (Table 1), as Lüthje and Schwarz revealed that the *mphC* gene often occurs linked to the *msrA* gene in *S. aureus* isolates [68,69,70,71]. Both the *mph* and *mef* genes encode macrolide phosphotransferase and macrolide efflux, respectively, which mediate specific resistance to macrolides [72].

Regarding *lnu* genes, they inactivate lincosamides by different types of lincosamide nucleotidyltransferases [73]. Among *lnuA*-carrying isolates, 42.9% had an iMLS_B_ phenotype, 42.9% had a cMLS_B_ phenotype, and 14.3% had an MS phenotype (Table 1). The prevalence of *lnuA* (13.5%) is comparable to that detected in China (18.4%) [74]. The *lnuB* gene was not detected among the tested isolates, which is in accordance with a study reported in Spain [75].

Screening for the inhibitory effect of the different compounds on erythromycin resistance against tested iMLS_B_ isolates indicated that doxorubicin and neomycin significantly decreased (*p*-value < 0.05) the MIC of erythromycin (Figure 4, Table 2 and Table S2). In addition, the checkerboard method revealed that doxorubicin and neomycin showed synergistic effects when combined with erythromycin (Table 4). Doxorubicin, a DNA-methyltransferase inhibitor [48], can compete with sinefungin, a SAM-competitive inhibitor and a methyltransferase inhibitor [76,77,78] at the SAM-binding site of the ErmC’ protein (Figure 5, Table S5, Figure S1). This inhibits the methylation of the macrolide binding site of 23S rRNA in the 50S subunit. As a result, erythromycin can attach to the ribosome and prevent protein synthesis [79]. Although doxorubicin can bind to the SAM-binding site of the ErmC’ protein, it showed a weak inhibitory activity on inducible clindamycin resistance. According to Svetlov and co-authors, erythromycin binding to ribosomes depends on its ability to form a hydrogen bond with the adenine nucleotide at position 2058 of the 23S rRNA, which is crucial for its activity [80]. Unlike erythromycin, clindamycin forms many hydrogen bonds with numerous amino acids, including A2058, A2059, G2505, and A2503, and van der Waal bonds with C2452 and U2506. This is why *N*-6-methylation of A2058 is not a key factor when it comes to clindamycin binding to the 23S rRNA of the ribosome [81]. Meanwhile, both erythromycin and neomycin act as protein synthesis inhibitors as neomycin attaches to the 30S ribosomal subunit [82,83]. In addition, neomycin is a positively charged antibiotic that interacts with negative charges in the RNA at multiple distant sites, enabling the stabilization and preventing the unfolding of RNA structures [84].

Additionally, neomycin and omeprazole significantly reduced the MIC of erythromycin against isolates no. 36 and 38 (MS phenotype) (Figure 4, Table 2 and Table S3). They also possessed synergistic effects with FICI ≤ 0.5 (Table 4). This may be due to the interactions between both compounds and the MsrA protein (Figure 5, Table S6, Figure S1). The latter is an ABC transporter protein, which removes macrolides and streptogramin B from the cell, preventing them from reaching their target site on the ribosome [85]. Docking analysis showed that neomycin and omeprazole were capable of binding to the MsrA protein, competing with erythromycin at the active site of MsrA protein, blocking the release of erythromycin out of *S. aureus*, and allowing erythromycin to exert its inhibitory effect on protein synthesis [86]. In addition, the lipophilic nature of omeprazole plays an important role in its solubility in bacterial membranes and its ability to bind to MsrA protein [87,88]. Furthermore, neomycin showed the highest binding affinity, suggesting that neomycin had the highest inhibitory effect on erythromycin resistance compared to omeprazole.

In addition, quinine showed a 4–8-fold decrease in the MIC of clindamycin against tested iMLS_B_ isolates (Table 3 and Table S4). In addition, quinine decreased the FICI of the clindamycin/erythromycin combination from 16.668 (antagonistic) to 0.56 (additive) against isolate no. 10 (Table 5). This effect may be related to the ability of quinine to bind to the ErmC’ protein (Figure 6 and Figure S2).

Additionally, the MIC of clindamycin was significantly lowered upon adding ketoprofen against all selected isolates (Table 3 and Table S4). Ketoprofen decreased FICI_DA/E_ from 16.668 to 0.75 against isolate no. 10 (Table 5). There may be two reasons for their inhibitory effect on inducible clindamycin resistance: (i) ketoprofen fit at the SAM-binding site of ErmC’, as detected by docking analysis (Figure 6 and Figure S2). Furthermore, ketoprofen is considered a thiopurine S-methyltransferase inhibitor via non-competitive inhibition [42]; (ii) as previously detected, ketoprofen inhibited the adherence of *S. aureus* [40,41], which could be the cause of its synergistic effect with clindamycin (Table 5).

Moreover, a 2–4-fold reduction in the MIC of clindamycin was observed after the addition of fosfomycin against all tested isolates (Table 3 and Table S4). On the other hand, an additive effect was observed when fosfomycin was combined with clindamycin and erythromycin, with FICI_DA/E_ falling from 16.668 to 0.56, as shown in Table 5. The good binding affinity of fosfomycin to ErmC’ (Figure 6 and Figure S2) and the synergism observed between fosfomycin and clindamycin (Table 5) could represent an explanation for the inhibitory effect of fosfomycin on inducible clindamycin resistance. The synergistic effect of the fosfomycin/clindamycin combination may be due to the cell wall inhibiting activity of fosfomycin that facilitates the entry of clindamycin into the bacterial cell, which in turn prevents the synthesis of bacterial proteins by targeting the 23S rRNA of the bacterial ribosome [89].

Molecular modeling studies showed that quinine, ketoprofen, and fosfomycin formed *H*-bond and hydrophobic interactions with key amino acids at the SAM-binding site of the ErmC’ protein (Table S7). This binding inhibits *N*-6 methylation of A2058 at the macrolides-binding site of 23S rRNA in 50S ribosomal subunit, thus allowing clindamycin to bind to its binding site on the ribosome and decreasing inducible clindamycin resistance [90]. In brief, quinine achieved the highest binding affinity, indicating that quinine had the highest inhibitory activity on inducible clindamycin resistance. Conversely, fosfomycin had the lowest effect.

## 4. Materials and Methods

### 4.1. Isolation and Identification of S. aureus Clinical Isolates

A total of 99 staphylococcal isolates were collected from different clinical sources (blood, wounds, nasal swabs, and broncho-alveolar lavage) over a period of 7 months from July 2018 to January 2019 from different hospitals near Mansoura University, Egypt. The study was approved by the Research Ethics Committee, Faculty of Pharmacy, Mansoura University (code no.: 2022-194). Standard procedures were followed for the isolation and identification of *S. aureus* from clinical specimens [91]. The purified *S. aureus* specimens were deposited as glycerol stocks at collection number MRCC6423 at the Microbial Resistant Culture Collection, Mansoura University, Egypt.

### 4.2. Antimicrobial Susceptibility Testing

The antimicrobial susceptibility of *S. aureus* clinical isolates to different classes of antimicrobial agents, including amoxicillin/clavulanic acid (30 μg), oxacillin (1 μg), cefoxitin (30 μg), ceftazidime (30 μg), cefotaxime (30 μg), cefepime (30 μg), imipenem (10 μg), gentamicin (10 μg), amikacin (30 μg), ciprofloxacin (5 μg), levofloxacin (5 μg), erythromycin (15 μg), azithromycin (15 μg), clarithromycin (15 μg), clindamycin (2 μg), quinapristin/dalfopristin (15 μg), and linezolid (30 μg) was evaluated by the Kirby–Bauer disk diffusion method using Mueller–Hinton agar plates (Oxoid, Thermo Fisher, Basingstoke, UK), according to the criteria set by the Clinical and Laboratory Standards Institute (CLSI) [92]. Isolates with an inhibition zone diameter ≤ 21 mm with cefoxitin and an inhibition zone diameter ≤ 10 mm with oxacillin were identified as MRSA [92]. Moreover, the MIC of vancomycin was detected by the broth microdilution method using Mueller–Hinton broth microtitre plates (Oxoid, Thermo Fisher, Basingstoke, UK), according to CLSI [92].

According to the definitions proposed by Magiorakos et al. [93], isolates that showed resistance to at least one agent in three or more antimicrobial classes were considered MDR. Extensive drug resistance (XDR) was identified as resistance to at least one agent in all antimicrobial classes except two or fewer. Furthermore, pandrug resistance (PDR) was identified as resistance to all agents in all antimicrobial classes.

### 4.3. Phenotypic Detection of MLS_B_ Phenotypes

The Mueller–Hinton agar plate was inoculated with an overnight culture of *S. aureus* isolates diluted to 0.5 McFarland. For detection of inducible clindamycin resistance, an erythromycin (15 μg) disk was placed at a distance of 15 mm (edge to edge) from the clindamycin (2 μg) disk. Then, these plates were incubated at 37 °C for 24 h.

Different MLS_B_-resistance phenotypes were detected among *S. aureus* isolates [94]: (i) cMLS_B_ phenotypes, including the R phenotype, where isolates were resistant to both erythromycin and clindamycin, and the HD phenotype, in which there were two growth zones around the clindamycin disk, one zone with a light hazy growth up to the clindamycin disk and the other with heavy growth and showed “D”. (ii) iMLS_B_ phenotypes, including the D phenotype, which showed a clear D zone around the clindamycin disk, and the D^+^ phenotype, in which tiny colonies were growing towards the clindamycin disk inside the D zone. (iii) The MS phenotype, in which isolates were sensitive to clindamycin but resistant to erythromycin without a D zone.

### 4.4. Detection of MLS_B_ Determinants by PCR

The detection of 18 genes, including *erm* genes (*ermA*, *ermB*, and *ermC*), *msr* genes (*msrA* and *msrB*), *lnu* genes (*lnuA* and *lnuB*), and *mph* genes (*mphC*) was performed by PCR reaction among MLS_B_-resistant *S. aureus* isolates.

The PCR was carried out as indicated: 12.5 μL DreamTaq Green PCR Master Mix (2X) (Thermo Fisher Scientific, Waltham, MA, USA), 2 μL of bacterial DNA (10 pg–1 μg), 1 μL of each primer (Table S8), and 8.5 μL nuclease-free water (Thermo Fisher Scientific, Waltham, MA, USA), for a total of 25 μL per reaction. The PCR protocol began with an initial denaturation of DNA at 95 °C for 5 min. Thereafter, there were 35 cycles of denaturation at 95 °C for 30 s, annealing at temperatures indicated for each primer as listed in Table S1 for 30 s, and extension at 72 °C for 1 min. Finally, there was an extension step at 72 °C for 5 min. The negative control was the reaction without the DNA template. A gel documentation system (Model Gel Documentation 1.4, 1189, AccuLab, New York, USA) was used to visualize PCR products after electrophoresis using 2% *w*/*v* agarose gel stained with ethidium bromide and compared visually with 100 base plus DNA marker (Thermo Fisher Scientific, Waltham, MA, USA) [95].

### 4.5. Screening for the Effect of Different Compounds on Erythromycin Resistance

Twenty-one different compounds were tested, including efflux inhibitor (CCCP), natural compounds (caffeine, quinine, quinidine, emetine, and piperine), antimicrobial agents (fosfomycin, neomycin, ciprofloxacin, and cancidas), chemotherapeutic agents (doxorubicin, 5-fluorouracil, and cisplatin), NSAIDs (meloxicam and ketoprofen), a proton pump inhibitor (omeprazole), vitamins (vitamin D3 and vitamin K), antihypertensive drugs (verapamil and digoxin), and a sedative drug (diazepam) (Table S9). Erythromycin thiocyanate was kindly provided from ADWIA, Egypt. The other compounds were supplied by Sigma-Aldrich, St. Louis, MO, USA.

The MIC of erythromycin and the tested twenty-one compounds were determined by the broth microdilution method against selected *S. aureus* isolates no. 10 (D phenotype) and no. 36 (MS phenotype) [92]. Two-fold serial dilutions of erythromycin and tested compounds were prepared in a 96-well microtiter plate. The diluted overnight culture of 1.5 × 10^5^ CFU/well was added to each well. The MIC values of erythromycin alone and in the presence of sub-inhibitory concentrations of the tested compounds were detected. The plates were incubated for 24 h at 37 °C. Wells containing culture with and without each tested compound were considered positive controls, while wells containing only broth were considered negative controls.

The compounds that exhibited inhibitory activity on erythromycin resistance against isolate no. 10 were further evaluated against isolates no. 14 and 27 (D phenotype) and no. 13 and 28 (D^+^ phenotype). Moreover, the compounds that elicited an inhibitory effect on erythromycin resistance against isolate no. 36 were similarly assessed against isolate no. 38 (MS phenotype).

### 4.6. Screening for the Effect of Different Compounds on Inducible Clindamycin Resistance

Isolate no. 10 (D phenotype) was selected for the preliminary screening of the activity of different tested compounds as potential inhibitors of inducible clindamycin resistance.

#### 4.6.1. Induction of Clindamycin Resistance by Erythromycin

Inducible clindamycin resistance was determined by the modified broth microdilution method according to CLSI [92]. The MIC of clindamycin was assessed alone and in the presence of a fixed concentration of erythromycin (8 μg/mL) against isolate no. 10. In brief, the two-fold serial dilutions of clindamycin alone or with erythromycin (8 μg/mL) were inoculated with a diluted culture of a final concentration of 1.5 × 10^5^ CFU/well. Thereafter, the plates were incubated for 24 h at 37 °C. Positive controls (inoculum with and without erythromycin) and negative controls (only media) were included in each plate.

#### 4.6.2. The Effect of Tested Compounds on Erythromycin-Induced Resistance to Clindamycin

The inhibitory activity of the tested compounds on inducible clindamycin resistance was determined using the broth microdilution method [92,96]. Determination of the MIC of clindamycin (in the presence of erythromycin (8 μg/mL)) was then repeated with the addition of a sub-inhibitory concentration of each tested compound. The plates were inoculated with diluted overnight culture (1.5 × 10^5^ CFU/well) of isolate no. 10. After that, plates were incubated at 37 °C for 24 h, including both positive and negative controls.

The compounds that showed inhibitory activity on inducible clindamycin resistance against isolate no. 10 were selected, and their activities were similarly evaluated against isolates no. 14 and 27 (D phenotype) and no. 13 and 28 (D^+^ phenotype).

### 4.7. Checkerboard Microdilution Method

Double combinations of erythromycin with potential inhibitors were tested against *S. aureus* isolate no. 10 (D phenotype) using the checkerboard microdilution method. Moreover, erythromycin was also combined with potential inhibitors against *S. aureus* isolate no. 36 (MS phenotype) in order to test the activity of these potential inhibitors on erythromycin resistance.

At the same time, three groups of combinations were performed, including (i) clindamycin with erythromycin, (ii) clindamycin with potential inhibitors, and (iii) clindamycin/erythromycin (8 μg/mL) with potential inhibitors against *S. aureus* isolate no. 10 (D phenotype) to evaluate the activity of these potential inhibitors on inducible clindamycin resistance.

Briefly, two-fold serial dilutions of antimicrobial agent (AMA) and potential inhibitor (PI) were prepared in sterile tubes. The concentrations used for each compound ranged from 1/8- to 4-fold the estimated MIC. Then, 50 μL of AMA was mixed with 50 μL of PI in a 96-well microtiter plate. Positive and negative controls were prepared for each combination. Finally, the plates were inoculated with a diluted culture of 1.5 × 10^5^ CFU/well and incubated for 24 h at 37 °C [97,98].

To evaluate the effect of the combinations, the MIC and FIC were calculated after overnight incubation. The combined effects were then determined based on FICI. For the combination of AMA and PI, FICI is calculated according to the following equation: FICI = FIC_AMA_ + FIC_PI_, where FIC_AMA_ = MIC_AMA (in the presence of PI)_/MIC_AMA(alone)_, and FIC_PI_ = MIC_PI (in the presence of AMA)_/MIC_PI(alone)_. AMA was either clindamycin or clindamycin/erythromycin (8 μg/mL).

According to the European Committee on Antimicrobial Susceptibility Testing (EUCAST) [99], the data were assessed as follows: a synergistic effect is observed when FICI ≤ 0.5; an additive effect is observed when 0.5< FICI ≤ 1; an indifferent effect is observed when FICI is between 1 and 2; and an antagonistic effect is present when FICI ≥ 2.

### 4.8. Docking Studies

The molecular docking calculations were performed as in the literature [100] using the Molecular Operating Environment (MOE) version 2019.01 Chemical Computing Group Inc. software [101]. The crystallographic structure of erythromycin-resistance methyltransferase (ErmC’) was obtained from the RCSB Protein Data Bank (PDB) (entry 1QAQ) and (entry 1QAO) [79]. The gene encoding macrolide-streptogramin resistance protein (MsrA) was obtained and downloaded as predicted Alpha fold UniProtKB (entry Q9ZNK9) [102]. Both MsrA and ErmC’ were prepared for molecular docking by the 3D-protonation and energy minimization using the MOE software 2009. Additionally, the energy-minimized structure served as a docking receptor. The site finder algorithm in MOE was utilized to identify SAM-binding site. ChemBioOffice was used to create the two-dimensional structures of the synthesized compounds, which were built using fragment libraries in MOE 2019, and energy was minimized using the MMFF94x force field until a root-mean-square (RMS) gradient of 0.01 kcal/mol was achieved. Redocking of co-crystallized ligands at binding sites was utilized to validate the docking setup and to show that it was appropriate for the intended docking study, which is supported by the small root-mean-square deviation (RMSD) of 1.43 Å (<2 Å) between the docked poses and the co-crystallized ligands. In order to identify and assess the interactions between ligands and the SAM-binding site of ErmC’, docking was carried out with specific parameters (rescoring function 1 and rescoring function 2: London dG, placement: triangle matcher, retain: 2, and refinement: force field). Based on the S-score of sinefungin and RMSD values, the most effective hits were chosen. The retrieved compounds that have lower RMSD and higher S-values were recorded.

### 4.9. Statistical Analysis

GraphPad Prism 5 (GraphPad Software Incorporation, San Diego, CA, USA) was adopted for all statistical analyses. The two-tailed paired t-test was used to compare two groups when one group represents the effect before adding a potential inhibitor and the other group indicates the effect after its addition. Probability values (*p*-values) <0.05 were considered statistically significant.

## 5. Conclusions

Our results revealed a high prevalence of erythromycin resistance (57.1%). In addition, isolates with inducible resistance phenotypes were also detected among tested isolates (14.3%). This confirms the importance of identifying potential inhibitors of erythromycin and inducible clindamycin resistance. Doxorubicin, neomycin, and omeprazole decreased the MIC of erythromycin significantly. Moreover, they showed synergistic effects when combined with erythromycin. On the other hand, quinine, ketoprofen, and fosfomycin caused a noticeable decrease in the MIC of clindamycin. Furthermore, quinine and fosfomycin decreased FICI_DA/E_ from 16.668 to 0.56, while ketoprofen reduced FICI _DA/E_ from 16.668 to 0.75. All data were confirmed by docking analysis. Therefore, doxorubicin, neomycin, and omeprazole could be potential inhibitors of erythromycin resistance, while quinine, ketoprofen, and fosfomycin could be potential inhibitors of inducible clindamycin resistance. Further experiments are required to confirm the data by *in vivo* studies.

## Figures and Tables

**Table 1:** Genotypes of macrolide-lincosamide-streptogramin B (MLS_B_)-resistant *S. aureus* clinical isolates.

Isolate Number	Genes Present	Result of D Test
1, 3, 4, 5, 6, 7, 8, 9, 21, 22, 23, 24, 25, 26, 29, 30, 31, 33, 34, 35, 39, 40, 41, 42, 43, 44, 45, 46, 48, 50.	*ermC*	R
2, 37, 49	*ermB*	R
10	*ermA*	D
11, 15	*ermC*, *lnuA*	D^+^
12	*ermC*, *lnuA*	HD
13, 18, 28, 52, 16, 17, 19	*ermC*	D^+^
14	*ermC*	D
20, 32	*ermC*, *lnuA*	R
27	*ermA*, *lnuA*	D
36	*msrA*, *msrB*, *mphC*, *lnuA*	MS
38	*msrA*, *msrB*, *mphC*	MS
47	*ermA*	R
51	*ermB*	D^+^

**Table 2:** Effect of potential inhibitors on erythromycin resistance against *S. aureus* isolates no. 10, 14, and 27 (D phenotype), isolates no. 13 and 28 (D^+^ phenotype), and isolates no. 36 and 38 (MS phenotype).

Isolate No.	MIC of E (μg/mL)	The Effect of Potential Inhibitors on Erythromycin Resistance					
		Potential Inhibitor	MICs (μg/mL)	Conc. Of the Inhibitor (μg/mL)	MICs of E in Combination with Inhibitor (μg/mL)	Fold Change	*p*-Value
10 (D)	1750	Quinine	1250	625	437.5	4-fold (-)	0.003 *
14 (D)	875		1250	625	218.75	4-fold (-)	0.02 *
27 (D)	1750		1250	625	218.75	8-fold (-)	0.003 *
13 (D^+^)	1750		1250	625	218.75	8-fold (-)	0.003 *
28 (D^+^)	1750		1250	625	218.75	8-fold (-)	0.003 *
10 (D)	1750	Fosfomycin	2	1	437.5	4-fold (-)	0.02 *
14 (D)	875		2	1	437.5	2-fold (-)	0.2
27 (D)	1750		2	1	437.5	4-fold (-)	0.02 *
13 (D^+^)	1750		2	1	875	2-fold (-)	0.02 *
28 (D^+^)	1750		1	0.5	437.5	4-fold (-)	0.02 *
10 (D)	1750	Doxorubicin	8	4	218.75	8-fold (-)	0.0005 *
14 (D)	875		8	4	109.375	8-fold (-)	0.0005 *
27 (D)	1750		8	4	54.688	32-fold (-)	0.0001 *
13 (D^+^)	1750		8	4	109.375	16-fold (-)	0.0001 *
28 (D^+^)	1750		8	4	54.688	32-fold (-)	0.0001 *
10 (D)	1750	Neomycin	1	0.5	109.375	16-fold (-)	0.0005 *
14 (D)	875		2	1	6.836	128-fold (-)	<0.0001 *
27 (D)	1750		1	0.5	218.75	8-fold (-)	0.003 *
13 (D^+^)	1750		16	8	218.75	8-fold (-)	0.02 *
28 (D^+^)	1750		2	1	218.75	8-fold (-)	0.004 *
36 (MS)	100		64	32	0.781	128-fold (-)	<0.0001 *
38 (MS)	200		0.0625	0.3125	6.25	32-fold (-)	0.0001 *
10 (D)	1750	Meloxicam	2048	1024	437.5	4-fold (-)	0.003 *
14 (D)	875		1024	512	218.75	4-fold (-)	0.02 *
27 (D)	1750		2048	1024	437.5	4-fold (-)	0.003 *
13 (D^+^)	1750		2048	1024	218.75	8-fold (-)	0.003 *
28 (D^+^)	1750		2048	1024	218.75	8-fold (-)	0.003 *
36 (MS)	100		1024	512	12.5	8-fold (-)	0.003 *
38 (MS)	200		128	64	50	4-fold (-)	0.003 *
10 (D)	1750	Ketoprofen	3125	1562.5	218.75	8-fold (-)	0.003 *
14 (D)	875		3125	1562.5	54.688	16-fold (-)	0.0005 *
27 (D)	1750		3125	1562.5	218.75	8-fold (-)	0.003 *
13 (D^+^)	1750		3125	1562.5	218.75	8-fold (-)	0.003 *
28 (D^+^)	1750		3125	1562.5	218.75	8-fold (-)	0.003 *
36 (MS)	100		3125	1562.5	12.5	8-fold (-)	0.003 *
38 (MS)	200		1562.5	781.25	25	8-fold (-)	0.003 *
36 (MS)	100	CCCP	2	1	25	4-fold (-)	0.02 *
38 (MS)	200		2	1	50	4-fold (-)	0.02 *
36 (MS)	100	Caffeine	22,000	2750	25	4-fold (-)	0.02 *
38 (MS)	200		5500	2750	50	4-fold (-)	0.02 *
36 (MS)	100	Omeprazole	5000	2500	12.5	8-fold (-)	0.0005 *
38 (MS)	200		625	312.5	50	4-fold (-)	0.003 *

Isolate no.: number of isolates, MICs: minimum inhibitory concentrations, E: erythromycin, conc.: concentration, *p*-value: probability-value, (-): decrease, *: probability-value is <0.05, which is considered statistically significant.

**Table 3:** Effect of potential inhibitors on inducible clindamycin resistance against *S. aureus* isolates no. 10, 14 and 27 (D phenotype) and isolates no. 13 and 28 (D^+^ phenotype).

Isolate No.	ICR		Fold Change	Inhibition of ICR					
	DA (μg/mL)	DA/E (μg/mL)		Potential Inhibitor	MICs (μg/mL)	Conc. of Inhibitor (μg/mL)	MICs of DA/E in Combination with Inhibitor (μg/mL)	Fold Change	*p*-Value
10 (D)	0.0625	1	16-fold (+)	Quinine	1250	625	0.25	4-fold (-)	0.02 *
14 (D)	0.0625	1			1250	625	0.25	4-fold (-)	0.02 *
27 (D)	0.0625	1			1250	625	0.25	4-fold (-)	0.02 *
13 (D^+^)	0.0625	16	256-fold (+)		1250	625	2	8-fold (-)	0.003 *
28 (D^+^)	0.0625	16			1250	625	4	4-fold (-)	0.003 *
10 (D)	0.0625	1	16-fold (+)	Fosfomycin	2	1	0.25	4-fold (-)	0.02 *
14 (D)	0.0625	1			2	1	0.25	4-fold (-)	0.02 *
27 (D)	0.0625	1			2	1	0.5	2-fold (-)	0.02 *
13 (D^+^)	0.0625	16	256-fold (+)		2	1	4	4-fold (-)	0.003 *
28 (D^+^)	0.0625	16			1	0.5	4	4-fold (-)	0.003 *
10 (D)	0.0625	1	16-fold (+)	Ketoprofen	3125	1562.5	0.25	4-fold (-)	0.02 *
14 (D)	0.0625	1			3125	1562.5	0.25	4-fold (-)	0.02 *
27 (D)	0.0625	1			3125	1562.5	0.25	4-fold (-)	0.02 *
13 (D^+^)	0.0625	16	256-fold (+)		3125	1562.5	0.5	32-fold (-)	0.0001 *
28 (D^+^)	0.0625	16			3125	1562.5	0.5	32-fold (-)	0.0001 *

Isolate no.: number of isolate, ICR: inducible clindamycin resistance, MICs: minimum inhibitory concentrations, E: erythromycin, DA: clindamycin, (+): increase, conc.: concentration, (-): decrease, *p*-value: probability-value, *: probability-value is <0.05 which is considered statistically significant.

**Table 4:** Combined effect of erythromycin/potential inhibitors on *S. aureus* isolates no. 10 (D phenotype) and no. 36 (MS phenotype) using checkerboard assay.

Isolate No.	Potential Inhibitor	FICI		Combined Effect
10 (D)	Quinine	E/Q	0.625	Additive
	Fosfomycin	E/F	0.75	Additive
	Doxorubicin	E/D	0.5	Synergism
	Neomycin	E/N	0.5	Synergism
	Meloxicam	E/M	0.75	Additive
	Ketoprofen	E/K	0.625	Additive
36 (MS)	CCCP	E/CCCP	0.625	Additive
	Caffeine	E/C	0.531	Additive
	Omeprazole	E/O	0.5	Synergism
	Neomycin	E/N	0.313	Synergism
	Meloxicam	E/M	0.625	Additive
	Ketoprofen	E/K	0.625	Additive

Isolate no.: number of isolate, FICI: fractional inhibitory concentration index, E: erythromycin, D: doxorubicin, N: neomycin, M: meloxicam, CCCP: carbonyl cyanide m-chlorophenylhydrazine, C: caffeine, O: omeprazole, K: ketoprofen.

**Table 5:** Combined effect of clindamycin/erythromycin/potential inhibitors on *S. aureus* isolate no. 10 (D phenotype) using checkerboard assay.

Isolate No.	FICI and Combined Effect of DA/E	Potential Inhibitor	FICI		Combined Effect
Isolate no. 10	16.668 (antagonism)	Quinine	DA/Q	1	Additive
			DA&E/Q	0.56	Additive
		Fosfomycin	DA/F	0.375	Synergism
			DA&E/F	0.56	Additive
		Ketoprofen	DA/K	0.5	Synergism
			DA&E/K	0.75	Additive

Isolate no.: number of isolate, FICI: fractional inhibitory concentration index, DA: clindamycin, E: erythromycin, Q: quinine, F: fosfomycin, K: ketoprofen.

## Data Availability

All data generated or analyzed during this study are included in this published article and its supplementary information files. Other datasets generated during and/or analyzed during the current study are available from the corresponding author upon reasonable request.

## Supplementary Materials

The following supporting information can be downloaded at: https://www.mdpi.com/article/10.3390/antibiotics12030503/s1, Figure S1: The 2D interactions of sinefungin and doxorubicin at S-adenosyl-L-methionine (SAM) binding site of ErmC’ protein, and erythromycin, neomycin, and omeprazole at MsrA protein; Figure S2: The 2D interactions of SAM, quinine, ketoprofen, and fosfomycin at SAM-binding site of ErmC’ protein; Table S1: Antimicrobial susceptibility of tested *S. aureus* clinical isolates to different classes of antimicrobial agents; Table S2: Effect of different tested compounds on erythromycin resistance against *S. aureus* isolate no. 10 (D phenotype); Table S3: Effect of different tested compounds on erythromycin resistance against *S. aureus* isolate no. 36 (MS phenotype); Table S4: Effect of different tested compounds on inducible clindamycin resistance against *S. aureus* isolate no. 10 (D phenotype); Table S5: Docking results for sinefungin and doxorubicin binding at ErmC’ protein (PDB ID: 1QAQ); Table S6: Docking results for erythromycin, neomycin and omeprazole binding at MsrA protein (UniProtKB ID: Q9ZNK9); Table S7: Docking results for S-adenosyl-L-methionine (SAM), quinine, ketoprofen, and fosfomycin binding at ErmC’ protein (PDB ID: 1QAO). Table S8: Primers used for detection of macrolide-lincosamide-streptogramin B (MLS_B_)-resistance determinants; Table S9: Different compounds screened for their activity on erythromycin resistance and inducible clindamycin resistance. References [103,104,105,106,107,108,109,110,111] are cited in the supplementary materials.
