# Supplementary material for: Inhibition of Erythromycin and Erythromycin-Induced Resistance among Staphylococcus aureus Clinical Isolates
Source: Antibiotics (Basel). 2023 Mar 2;12(3):503. doi: 10.3390/antibiotics12030503 (PMC10044026; doi:10.3390/antibiotics12030503)
Supplement: Supplementary file 1 [file antibiotics-12-00503-s001.zip › antibiotics-2205979-supplementary.pdf]

Antibiotics

Inhibition of Erythromycin and Erythromycin-Induced Resistance Among *Staphylococcus aureus* Clinical Isolates

Aya A. Mahfouz [a,c], Heba S. Said [a], Sherin M. Elfeky [b], Mona I. Shaaban [a*]

[a] Department of Microbiology and Immunology, Faculty of Pharmacy, Mansoura University, Mansoura 35516, Egypt.

[b] Department of Pharmaceutical Organic Chemistry, Faculty of Pharmacy, Mansoura University, Mansoura 35516, Egypt.

[c] Department of Microbiology and Immunology, Faculty of Pharmacy, Horus University-Egypt, New Damietta 34518, Egypt.

* Corresponding author: Mona I. Shaaban

E-mail addresses: mona_ibrahem@mans.edu.eg; sarafawzy2002@yahoo.com

Telephone: 01066944268

Table S1. Antimicrobial susceptibility of tested *S. aureus* clinical isolates to different classes of antimicrobial agents.

Isolate no.	Clinical Source	β-lactams							Aminoglycosides		Fluoroquinolones		Macrolides			Lincosamides	Streptogramins	Oxazolidinones	MRSA or MSSA
		AMC	OX	Fox	CAZ	CTX	FEP	IPM	CN	AK	CIP	LEV	CLR	AZM	E	DA	RP	LZD	
1	Wound	R	R	R	R	R	R	R	R	R	R	R	R	R	R	R	R	S	MRSA
2	Wound	R	R	R	R	R	R	S	R	R	R	R	R	R	R	R	R	S	MRSA
3	Wound	R	R	R	R	R	R	S	R	S	S	S	R	R	R	R	R	S	MRSA
4	Wound	R	R	R	R	R	R	R	R	R	R	R	R	R	R	R	R	S	MRSA
5	Wound	R	R	R	R	R	R	R	R	R	R	R	R	R	R	R	R	S	MRSA
6	Wound	R	R	R	R	R	R	R	R	R	R	R	R	R	R	R	R	S	MRSA
7	Wound	R	R	R	R	R	R	R	R	R	R	R	R	R	R	R	R	S	MRSA
8	Wound	R	R	R	R	R	R	R	R	R	R	R	R	R	R	R	R	S	MRSA
9	Wound	R	R	R	R	R	R	S	R	I	R	R	R	R	R	R	R	S	MRSA
10	Wound	R	R	R	R	R	R	S	S	S	S	S	R	R	R	S	R	S	MRSA
11	Wound	R	S	R	R	I	I	S	R	S	R	S	R	R	R	S	R	S	MRSA
12	Wound	R	I	R	R	R	R	S	R	R	R	S	R	R	R	S	R	S	MRSA
13	Wound	R	R	R	R	R	R	S	R	S	S	S	R	R	R	S	R	S	MRSA
14	Wound	R	R	R	R	I	I	S	S	S	R	R	R	R	R	S	R	S	MRSA
15	Wound	R	R	R	R	I	R	S	S	S	S	S	R	R	R	S	R	S	MRSA
16	Wound	R	R	R	R	R	R	R	S	S	R	R	R	R	R	S	R	S	MRSA
17	Wound	R	R	R	R	R	R	S	R	S	S	S	R	R	R	S	R	S	MRSA
18	Wound	R	I	R	R	R	R	S	S	S	S	S	R	R	R	S	R	S	MRSA
19	Wound	R	I	R	R	R	R	S	S	S	S	S	R	R	R	S	R	S	MRSA
20	Blood	R	R	R	R	R	R	S	S	S	S	S	I	R	R	R	R	S	MRSA
21	Blood	R	R	R	R	R	R	R	R	R	R	R	R	R	R	R	R	S	MRSA
22	Blood	R	R	R	R	R	R	R	R	S	S	R	R	R	R	R	R	S	MRSA
23	Blood	R	R	R	R	R	R	S	R	S	R	S	R	R	R	R	R	S	MRSA
24	Blood	R	R	R	R	R	R	R	R	R	R	R	R	R	R	R	R	S	MRSA
25	Blood	R	R	R	R	R	R	R	R	S	R	R	R	R	R	R	R	S	MRSA
26	Blood	R	R	R	R	R	R	I	R	R	R	R	R	R	R	R	R	S	MRSA
27	Blood	R	R	R	R	R	R	S	S	S	I	S	R	R	R	S	R	S	MRSA
28	Skin tissue	R	R	R	R	R	R	S	R	R	S	S	R	R	R	S	R	S	MRSA
29	Nasal swab	R	R	R	R	R	R	R	R	R	R	R	R	R	R	R	R	S	MRSA

30	**Nasal swab**	**R**	**R**	**R**	**R**	**R**	**R**	**R**	**R**	**R**	**R**	**R**	**R**	**R**	**R**	**R**	**R**	**S**	**MRSA**
31	Nasal swab	R	R	R	R	R	R	R	R	R	R	R	R	R	R	R	R	S	MRSA
32	Nasal swab	R	R	R	R	S	R	R	R	R	R	R	R	R	R	R	R	S	MRSA
33	Nasal swab	R	R	R	R	S	R	R	R	R	R	R	R	R	R	R	R	S	MRSA
34	Nasal swab	R	R	R	R	R	R	R	R	R	R	R	R	R	R	R	R	S	MRSA
35	Nasal swab	R	R	R	R	R	R	R	R	R	R	R	R	R	R	R	R	S	MRSA
36	Nasal swab	R	R	R	R	R	R	R	R	R	R	S	R	R	R	S	R	S	MRSA
37	Nasal swab	S	R	R	R	R	R	R	R	S	R	S	R	R	R	R	R	S	MRSA
38	Nasal swab	S	S	S	R	R	S	S	S	S	S	S	R	R	R	S	R	S	MSSA
39	Nasal swab	S	R	R	R	I	S	S	R	S	S	S	R	R	R	R	R	S	MRSA
40	Nasal swab	R	R	R	R	I	R	S	S	S	S	S	R	R	R	R	R	S	MRSA
41	Nasal swab	S	R	R	R	R	S	S	R	R	R	R	R	R	R	R	R	S	MRSA
42	Nasal swab	R	R	R	R	R	R	R	R	R	R	R	R	R	R	R	R	S	MRSA
43	Nasal swab	S	R	R	R	R	S	S	S	S	S	S	R	R	R	R	R	S	MRSA
44	Nasal swab	R	R	R	R	I	R	R	R	R	R	R	R	R	R	R	R	S	MRSA
45	Nasal swab	S	R	R	R	R	R	S	R	S	I	S	R	R	R	R	R	S	MRSA
46	Nasal swab	R	R	R	R	R	R	I	R	R	R	R	R	R	R	R	R	S	MRSA
47	Nasal swab	R	R	R	R	R	R	R	R	R	R	R	R	R	R	R	R	S	MRSA
48	Nasal swab	R	R	R	R	R	R	S	R	R	R	R	R	R	R	R	R	S	MRSA
49	Nasal swab	S	R	R	R	R	I	S	S	S	R	R	R	R	R	S	R	S	MRSA
50	Nasal swab	R	R	R	R	R	I	S	R	S	S	S	R	R	R	R	R	S	MRSA
51	Nasal swab	S	S	R	R	R	I	S	I	S	R	I	R	R	R	S	R	S	MRSA
52	Nasal swab	R	R	R	R	R	R	S	S	S	I	S	R	R	R	S	R	S	MRSA
53	Wound	R	R	R	R	R	R	S	R	R	S	S	S	S	S	S	S	S	MRSA
54	Wound	R	R	R	R	R	R	S	R	I	S	S	S	S	S	S	S	S	MRSA
55	Wound	R	R	R	R	I	I	S	R	S	S	S	S	S	S	S	S	S	MRSA
56	Wound	R	R	R	R	I	R	S	R	S	S	S	S	S	S	S	S	S	MRSA

57	Wound	R	R	R	R	I	I	S	S	S	S	S	S	S	S	S	S	S	MRSA
58	Wound	S	R	R	R	I	I	S	R	S	S	S	S	S	S	S	S	S	MRSA
59	Wound	R	R	R	R	R	R	S	S	S	I	S	S	S	S	S	S	S	MRSA
60	Wound	R	R	R	R	I	I	S	S	S	S	S	S	S	S	S	S	S	MRSA
61	Wound	S	S	S	S	S	S	S	S	S	S	S	S	S	S	S	S	S	MSSA
62	Wound	R	S	R	R	I	S	S	R	S	S	S	S	S	S	S	S	S	MRSA
63	Wound	R	S	R	R	I	S	S	R	R	I	S	S	S	S	S	S	S	MRSA
64	Wound	R	I	R	R	I	I	S	R	R	R	S	S	S	S	S	S	S	MRSA
65	Wound	R	S	R	R	I	I	S	R	S	S	S	S	S	S	S	S	S	MRSA
66	Wound	R	R	R	R	R	R	S	R	S	R	R	S	S	S	S	S	S	MRSA
67	Wound	R	R	R	R	I	R	S	R	R	R	S	S	S	S	S	S	S	MRSA
68	Wound	R	R	R	R	I	R	S	R	S	R	R	S	S	S	S	S	S	MRSA
69	Blood	R	S	R	R	I	S	S	R	R	S	S	S	S	S	S	S	S	MRSA
70	Blood	R	S	R	R	I	I	S	R	S	S	S	S	S	S	S	S	S	MRSA
71	Blood	R	S	R	I	I	I	S	R	R	S	S	S	S	S	S	S	S	MRSA
72	Blood	R	S	R	R	I	S	S	S	S	S	S	S	S	S	S	S	S	MRSA
73	Blood	R	R	R	R	I	I	S	S	S	S	S	S	S	S	S	S	S	MRSA
74	Blood	S	S	R	R	I	S	S	S	S	S	S	S	S	S	S	S	S	MRSA
75	Blood	R	S	R	R	I	I	S	S	S	S	S	S	S	S	S	S	S	MRSA
76	Blood	R	S	R	R	I	S	S	S	S	S	S	S	S	S	S	S	S	MRSA
77	Blood	R	S	S	S	S	S	S	S	S	S	S	S	S	S	S	S	S	MSSA
78	Blood	S	S	S	S	S	S	S	R	S	S	S	S	S	S	S	S	S	MSSA
79	Blood	R	S	R	R	R	R	S	R	S	S	S	S	S	S	S	S	S	MRSA
80	Blood	R	S	R	R	I	S	S	S	S	S	S	S	S	S	S	S	S	MRSA
81	Blood	R	R	R	R	R	R	R	R	R	R	R	S	S	S	S	S	S	MRSA
82	Blood	S	I	R	R	R	S	S	I	S	S	S	S	S	S	S	S	S	MRSA
83	Blood	S	S	R	R	R	S	S	R	S	S	S	S	S	S	S	S	S	MRSA
84	Blood	S	I	R	R	R	S	S	S	S	S	S	S	S	S	S	S	S	MRSA
85	Blood	S	R	R	R	R	S	S	S	S	S	S	S	S	S	S	S	S	MRSA
86	Blood	S	S	R	R	R	S	S	S	S	S	S	S	S	S	S	S	S	MRSA
87	Blood	R	R	R	R	R	R	R	R	R	R	R	S	S	S	S	S	S	MRSA
88	Blood	R	R	R	R	R	R	S	S	S	R	S	S	S	S	S	S	S	MRSA
89	Blood	S	S	S	R	R	S	S	S	S	S	S	S	S	S	S	S	S	MSSA
90	Blood	R	R	R	R	R	R	R	R	R	R	R	S	S	S	S	S	S	MRSA
91	B A L	R	S	R	R	S	S	S	S	S	S	S	S	S	S	S	S	S	MRSA

Isolate no.: the number of isolate, AMC: amoxicillin-clavulanic acid, OX: oxacillin, FOX: cefoxitin, CAZ: ceftazidime, CTX: cefotaxime, FEP: cefepime, IPM: imipenem, CN: gentamicin, AK: amikacin, CIP: ciprofloxacin, LEV: levofloxacin, CLR: clarithromycin, AZM: azithromycin, E: erythromycin, DA: clindamycin, RP: quinapristin/dalfopristin, LZD: linezolid, MRSA: methicillin resistant *S. aureus*, MSSA: methicillin sensitive *S. aureus*, MDR: multidrug-resistant, XDR: extensively drug-resistant, PDR: pandrug-resistant, S: susceptible, I: intermediate, R: resistant, BAL: broncho-alveolar lavage.

Table S2. Effect of different tested compounds on erythromycin resistance against *S. aureus* isolate no. 10 (D phenotype).

Isolate no.	MIC of E (µg/mL)	The effect of tested compounds on erythromycin resistance				
		**Tested compound**	**MICs (µg/mL)**	**Conc. of inhibitor (µg/mL)**	**MICs of E in combination with inhibitor (µg/mL)**	**Fold change**
Isolate no. 10	1750	CCCP	0.5	0.25	1750	—
		Caffeine	22,000	2,750	875	2-fold ⬇
		Quinine	1250	625	437.5	**4-fold** ⬇
		Quinidine	625	312.5	875	2-fold ⬇
		Emetine	6250	3125	875	2-fold ⬇
		Cancidase	97.656	48.828	875	2-fold ⬇
		Piperine	20,000	5000	875	2-fold ⬇
		Fosfomycin	2	1	437.5	**4-fold** ⬇
		Neomycin	1	0.5	109.375	**16-fold** ⬇
		Ciprofloxacin	0.25	0.125	875	2-fold ⬇
		Doxorubicin	8	4	218.75	**8-fold** ⬇
		5-Fluorouracil	0.5	0.25	875	2-fold ⬇
		Cisplatin	128	64	875	2-fold ⬇
		Meloxicam	2048	1024	437.5	**4-fold** ⬇
		Ketoprofen	3125	1562.5	218.75	**8-fold** ⬇
		Omeprazole	625	312.5	875	2-fold ⬇
		Vitamin D3	1024	512	875	2-fold ⬇
		Vitamin K	2500	1250	875	2-fold ⬇
		Verapamil	1024	512	875	2-fold ⬇
		Digoxin	62.5	31.25	875	2-fold ⬇
		Diazepam	256	128	875	2-fold ⬇

Table S3. Effect of different tested compounds on erythromycin resistance against *S. aureus* isolate no. 36 (MS phenotype).

**Isolate no.**	**MIC of E (μg/mL)**	**The effect of tested compounds on erythromycin resistance**				
		**Tested compound**	**MICs (μg/mL)**	**Conc. of inhibitor (μg/mL)**	**MICs of E in combination with inhibitor (μg/mL)**	**Fold change**
Isolate no. 36	100	CCCP	2	1	25	**4-fold ⬇**
		Caffeine	22,000	2,750	25	**4-fold ⬇**
		Quinine	625	312.5	100	__
		Quinidine	625	312.5	50	2-fold ⬇
		Emetine	6250	3125	50	2-fold ⬇
		Cancidase	58.594	29.297	100	__
		Piperine	20,000	5000	100	__
		Fosfomycin	4	2	50	2-fold ⬇
		Neomycin	64	32	0.781	**128-fold ⬇**
		Ciprofloxacin	32	16	50	2-fold ⬇
		Doxorubicin	4	2	50	2-fold ⬇
		5-Fluorouracil	0.125	0.0625	100	__
		Cisplatin	1000	500	100	__
		Meloxicam	1024	512	12.5	**8-fold ⬇**
		Ketoprofen	3125	1562.5	12.5	**8-fold ⬇**
		Omeprazole	5000	2500	12.5	**8-fold ⬇**
		Vitamin D3	1024	512	100	__
		Vitamin K	2500	1250	100	__
		Verapamil	625	312.5	50	2-fold ⬇
		Digoxin	62.5	31.25	100	__
		Diazepam	128	64	100	__

Isolate no.: number of isolate, E: erythromycin, conc.: concentration, ⬇: decrease.

Table S4. Effect of different tested compounds on inducible clindamycin resistance against *S. aureus* isolate no. 10 (D phenotype).

Isolate no.	ICR			Inhibition of ICR				
	DA (µg/mL)	DA/E (µg/mL)	Fold change	Tested compound	MICs (µg/mL)	Conc. of inhibitor (µg/mL)	MICs of DA/E in combination with inhibitor (µg/mL)	Fold change
Isolate no. 10	0.0625	1	16-fold⬆	CCCP	0.5	0.25	1	__
				Caffeine	22,000	2,750	0.5	2-fold⬇
				Quinine	1250	625	0.25	**4-fold⬇**
				Quinidine	625	312.5	0.5	2-fold⬇
				Emetine	6250	3125	1	__
				Cancidase	97.656	48.828	0.5	2-fold⬇
				Piperine	20,000	5000	0.5	2-fold⬇
				Fosfomycin	2	1	0.25	**4-fold⬇**
				Neomycin	1	0.5	0.5	2-fold⬇
				Ciprofloxacin	0.25	0.125	0.5	2-fold⬇
				Doxorubicin	8	4	0.5	2-fold⬇
				5-Fluorouracil	0.5	0.25	1	__
				Cisplatin	128	64	0.5	2-fold⬇
				Meloxicam	2048	1024	0.5	2-fold⬇
				Ketoprofen	3125	1562.5	0.25	**4-fold⬇**
				Omeprazole	625	312.5	0.5	2-fold⬇
				vitamin D3	1024	512	1	__
				Vitamin K	2500	1250	1	__
				Verapamil	1024	512	0.5	2-fold⬇
				Digoxin	62.5	31.25	0.5	2-fold⬇
				Diazepam	256	128	0.5	2-fold⬇

Isolate no.: number of isolate, ICR: inducible clindamycin resistance, E: erythromycin, DA: clindamycin, ⬆: increase, conc.: concentration, ⬇:decrease.

Table S5. Docking results for sinefungin and doxorubicin binding at ErmC` protein (PDB ID: 1QAQ).

Compound	Docking score (ΔG) (kcal/mol)	*H*-bond (length Å)	Hydrophobic interactions
**Sinefingin**	-9.75	Glu38 (2.96)<br>Glu59 (2.4, 2.65)<br>Asn101 (2.89, 2.55)<br>Ile85 (2.98)<br>Ile13 (3.02)<br>Asp84 (3.39)	Pro103<br>Asn101<br>Asn11<br>Gly38<br>Gln10<br>Ile60<br>Ile106
**Doxorubicin**	-7.59	Glu38 (2.62)<br>Glu59 (2.96)<br>Asn11 (2.84)<br>Ile58 (2.95)<br>Ile60 (3.29)<br>Gly40 (3.56)	Pro103<br>Asn101<br>Asn11<br>Gly38<br>Gln10<br>Ile60<br>Ile106<br>Ile85<br>Asp61<br>Ser9

Table S6. Docking results for erythromycin, neomycin and omeprazole binding at MsrA protein (UniProtKB ID: Q9ZNK9).

Compound	Docking score (ΔG) (kcal/mol)	*H*-bond (length Å)	Hydrophobic interactions
**Erythromycin**	-5.91	Glu449 (3.54)<br>Met450 (3.4)	Glu446<br>Met450<br>Tyr280<br>Gln189<br>Tyr192<br>Ile442<br>Glu193<br>Gln196<br>Leu197<br>His471<br>Glu282
**Neomycin**	-7.21	Glu449 (3.21)<br>Met450 (3.14)<br>Glu446 (3.23)<br>Lys443 (3.15)<br>Asp171 (3.44, 3.55)	Glu446<br>Met450<br>Tyr280<br>Gln189<br>Tyr192<br>Ile442<br>Glu193<br>Lys443
**Omeprazole**	-6.93	Glu446 (2.9)	Glu446<br>Tyr280<br>Tyr192<br>Ile442<br>Glu193<br>Gln196<br>Lys443<br>Gly38<br>Tyr188

Table S7. Docking results for S-adenosyl-L-methionine (SAM), quinine, ketoprofen, and fosfomycin binding at ErmC` protein (PDB ID: 1QAO).

Ligand	Docking score (ΔG) (kcal/mol)	*H*-bond (length Å)	Hydrophobic interactions
**SAM**	-8.58	Gly38 (2.66)<br>Glu59 (2.62, 2.44, 3.31)<br>lle85 (2.91)<br>lle13 (3.52)<br>Asn101 (2.95)<br>Asp84 (3.01)	Pro103<br>Gly38<br>Ile60<br>Gln10<br>Ile106<br>Asn11<br>Asn101
**Quinine**	-6.87	Gly38 (3.25)<br>Glu59 (3.1)	Pro103<br>Gly38<br>Ile60<br>Gln10<br>Ile106<br>Asn11<br>Asn101
**Ketoprofen**	-6.52	Ile85 (3.03)<br>Ile60 (3.43)	Pro103<br>Gly38<br>Ile60<br>Gln10<br>Ile106
**Fosfomycin**	-4.19	Ile13 (2.99)<br>Asn101 (2.95)<br>Lys41 (2.85)<br>Gly40 (3.84)<br>Phe44 (2.84)	–

Table S8. Primers used for detection of macrolide-lincosamide-streptogramin B (MLS_B)-resistance determinants.

Gene name		Type	Nucleotide Sequence (5' to 3')	AT	Amplicon size (bp)	Ref
**Erythromycin resistance methylase (*erm*) genes**	***ermC***	Fw	AATCGTCAATTCCTGCATGT	57°C	299	(1)
		Rv	TAATCGTGGAATACGGGTTTG			
	ermA	Fw	GTTCAAGAACAATCAATACAGAG	56°C	421	(2)
		Rv	GGATCAGGAAAAGGACATTTTAC			
	ermB	Fw	CGAAATTGGAACAGGTAAAGG	53°C	359	This study
		Rv	GAATCGAGACTTGAGTGTGC			
**Macrolide-streptogramin B resistance (*msr*) genes**	***msrA***	Fw	GGCACAATAAGAGTGTTTAAAGG	57°C	940	(3)
		Rv	AAGTTATATCATGAATAGATTGTCCTGTT			
	msrB	Fw	TATGATATCCATAATAATTATCCAATC	50°C	595	(3)
		Rv	AAGTTATATCATGAATAGATTGTCCTGTT			
**Lincosamide nucleotidyl-transferase (*lnu*) genes**	***lnuA***	Fw	GGTGGCTGGGGGGTAGATGTATTAACTGG	57°C	310	This study
		Rv	GCTTCTTTTGAAATACATGGTATTTTTCGATC			
	lnuB	Fw	CCTACCTATTGTTTGTGGAA	54°C	943	(4)
		Rv	ATAACGTTACTCTCCTATTC			
**Macrolide phospho-transferase (*mphC*) genes**	***mphC***	Fw	GAGACTACCAAGAAGACCTGACG	56°C	722	(5)
		Rv	CATACGCCGATTCTCCTGAT			

Fw: forward, Rv: reverse, AT: annealing temperature, bp: base pair, Ref: reference.

Table S9. Different compounds screened for their activity on erythromycin resistance and inducible clindamycin resistance.

Type of inhibitor	Name of inhibitor
Efflux pump inhibitors	CCCP
Natural compounds	Caffeine<br>Quinine<br>Quinidine<br>Emetine<br>Piperine
Antimicrobial agents	Fosfomycin<br>Neomycin<br>Ciprofloxacin<br>Cancidas
Chemotherapeutic agents	Doxorubicin<br>5-Fluorouracil<br>Cisplatin
NSAIDs	Meloxicam<br>Ketoprofen

Proton pump inhibitors	Omeprazole
Vitamins	Vitamin D3<br>Vitamin K
Antihypertensive drugs	Verapamil<br>Digoxin
Sedatives	Diazepam

CCCP: carbonyl cyanide m-chlorophenylhydrazine, NSAIDs: non-steroidal anti-inflammatory drugs.

(a) **(b)**

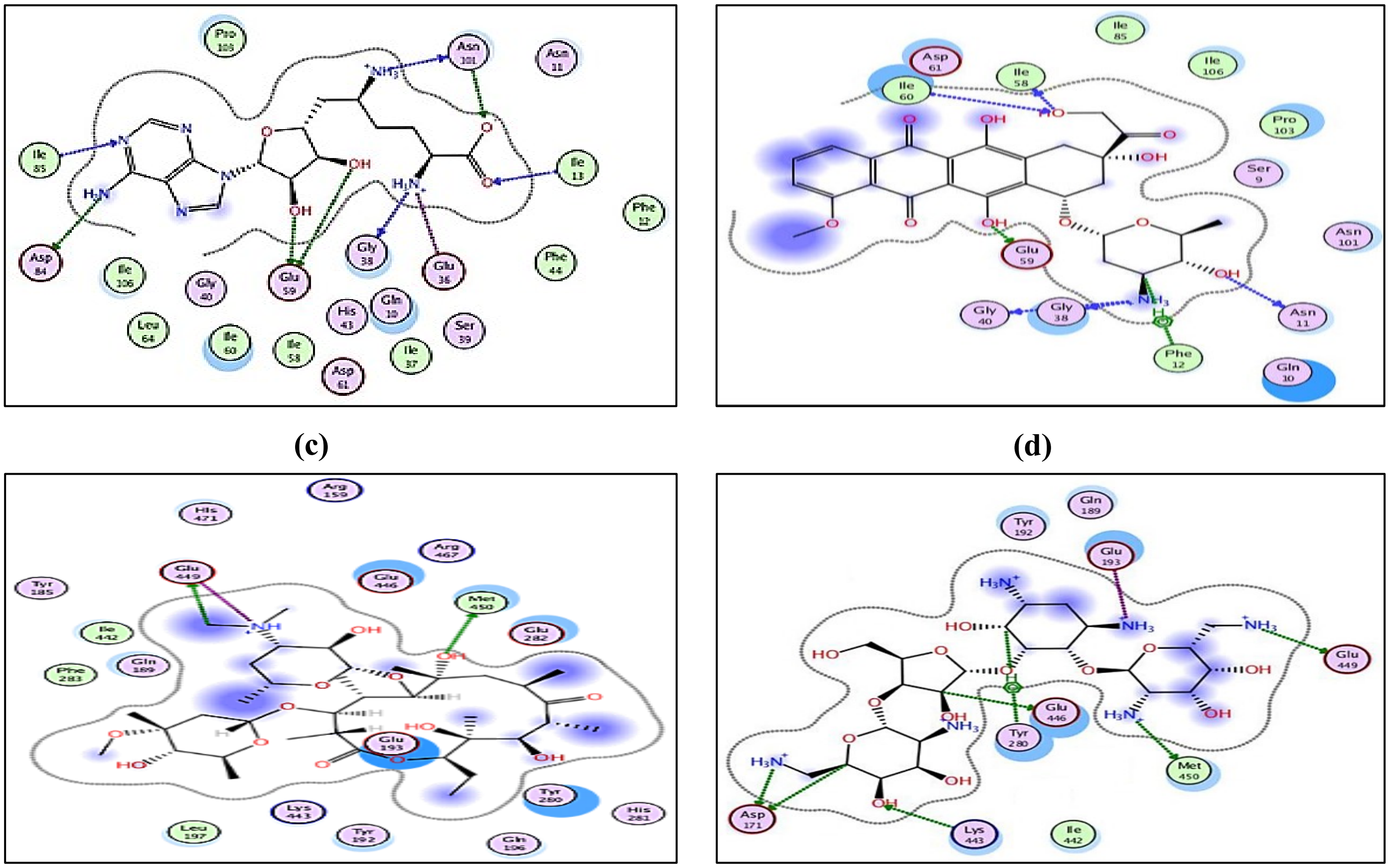

(e)

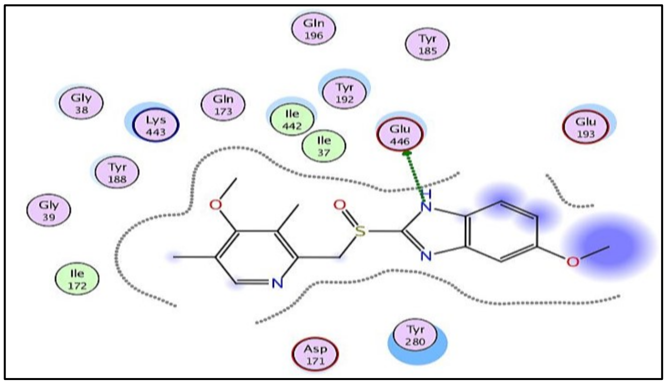

Figure S1. The 2D interactions of sinefungin and doxorubicin at S-adenosyl-L-methionine (SAM)-binding site of ErmC` protein, and erythromycin, neomycin, and omeprazole at MsrA protein. (a)** 2D diagram of sinefungin at SAM-binding site of ErmC` protein. **(b) 2D diagram of doxorubicin at SAM-binding site of ErmC`. **(c)** 2D representation of erythromycin at active site of MsrA protein. **(d)** 2D representation of neomycin at active site of MsrA. **(e)** 2D representation of omeprazole at active site of MsrA.

(a)

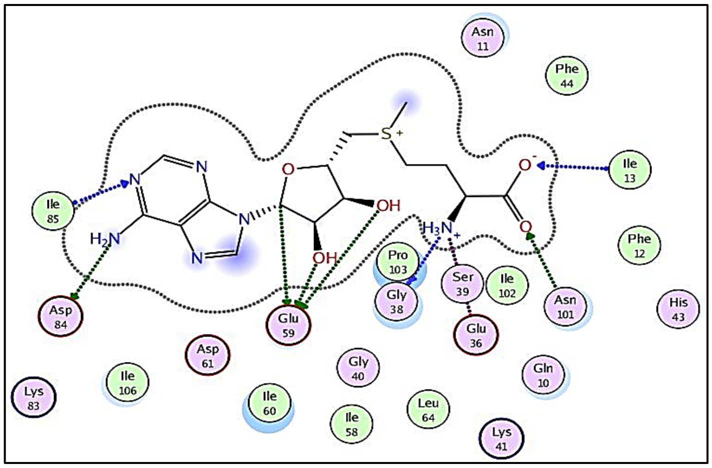

(b)

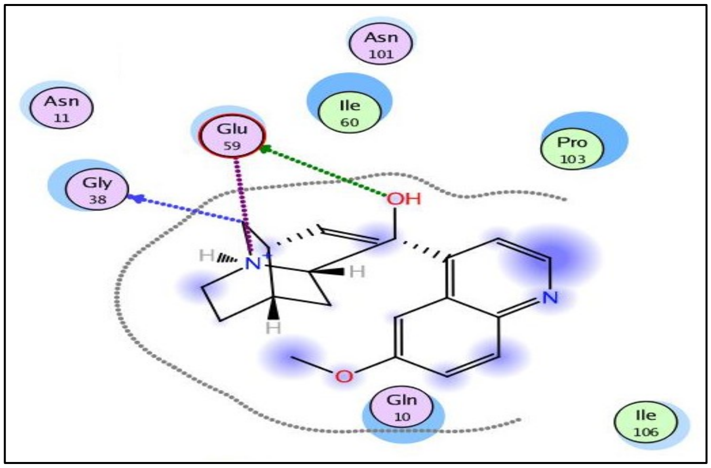

(c) (d)

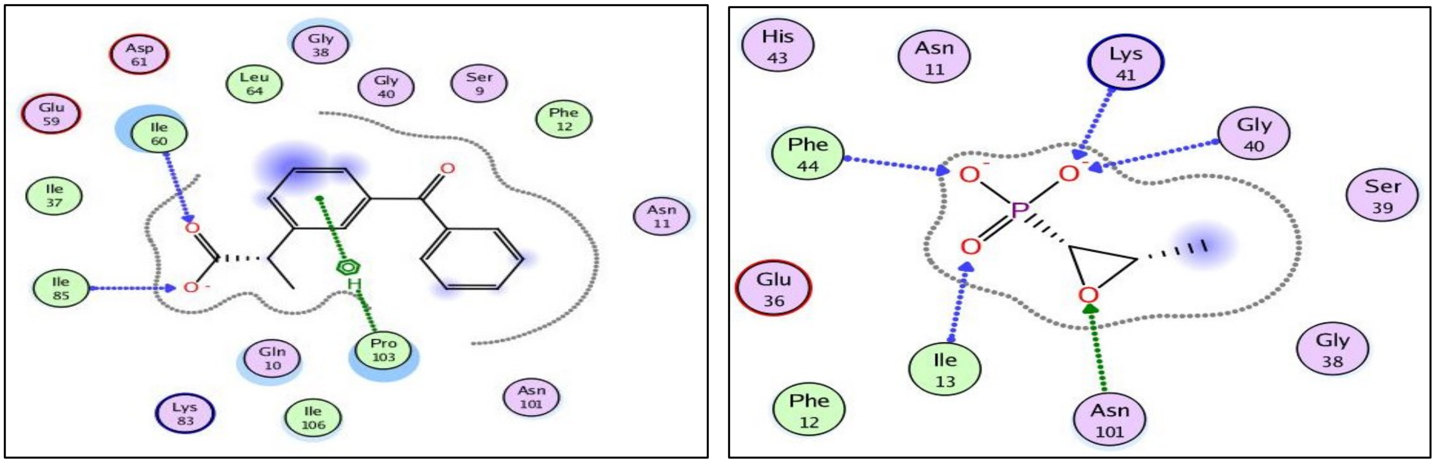

Figure S2. The 2D interactions of SAM, quinine, ketoprofen, and fosfomycin at SAM-binding site of ErmC` protein. (a)** 2D diagram of SAM at SAM-binding site of ErmC` protein. **(b) 2D diagram of quinine at SAM-binding site of ErmC`. **(c)** 2D diagram of ketoprofen at SAM-binding site of ErmC`. **(d)** 2D diagram of fosfomycin at SAM-binding site of ErmC`.

References

1. Strommenger, B.; Kettlitz, C.; Werner, G.; Witte, W. Multiplex PCR assay for simultaneous detection of nine clinically relevant antibiotic resistance genes in *Staphylococcus aureus*. *J. Clin. Microbiol.* **2003**, 41, 4089-94.
2. Leclercq, R.; Bauduret, F.; Soussy, C. Selection of constitutive mutants of gram-positive cocci inducible resistant to macrolides, lincosamides and streptogramins (MLS): comparison of the selective effects of the MLS. *Pathol. Biol.* **1989**, 37, 568-72.
3. Ross, J.; Eady, E.; Cove, J.; Cunliffe, W.; Baumberg, S.; Wootton, J. Inducible erythromycin resistance in staphlyococci is encoded by a member of the ATP-binding transport super-gene family. *Mol. Microbiol.* **1990**, 4, 1207-14.
4. Bozdogan, B.l.; Berrezouga, L.; Kuo, M.-S.; Yurek, D.A.; Farley, K.A.; Stockman, B.J., et al. A new resistance gene, *linB*, conferring resistance to lincosamides by nucleotidylation in *Enterococcus faecium* HM1025. *Antimicrob. Agents Chemother.* **1999**, 43, 925-9.

5. Lüthje, P.; Schwarz, S. Antimicrobial resistance of coagulase-negative staphylococci from bovine subclinical mastitis with particular reference to macrolide–lincosamide resistance phenotypes and genotypes. *J. Antimicrob. Chemother.* **2006**, 57, 966-9.
6. Sutcliffe, J.; Grebe, T.; Tait-Kamradt, A.; Wondrack, L. Detection of erythromycin-resistant determinants by PCR. *Antimicrob. Agents Chemother.* **1996**, 40, 2562-6.
7. Allignet, J.; Loncle, V.; El Solh, N. Sequence of a staphylococcal plasmid gene, *vga*, encoding a putative ATP-binding protein involved in resistance to virginiamycin A-like antibiotics. *Gene* **1992**, 117, 45-51.
8. Loncle, V.; Casetta, A.; Buu-Hoi, A.; El Solh, N. Analysis of pristinamycin-resistant *Staphylococcus epidermidis* isolates responsible for an outbreak in a Parisian hospital. *Antimicrob. Agents Chemother.* **1993**, 37, 2159-65.
9. Hammerum, A.M.; Jensen, L.B.; Aarestrup, F.M.l. Detection of the *satA* gene and transferability of virginiamycin resistance in *Enterococcus faecium* from food-animals. *FEMS Microbiol. Lett.* **1998**, 168, 145-51.
10. Allignet, J.; Liassine, N.; El Solh, N. Characterization of a staphylococcal plasmid related to pUB110 and carrying two novel genes, *vatC* and *vgbB*, encoding resistance to streptogramins A and B and similar antibiotics. *Antimicrob. Agents Chemother.* **1998**, 42, 1794-8.
